# Local regulation of the Srs2 helicase by the SUMO-like domain protein Esc2 promotes recombination at sites of stalled replication

**DOI:** 10.1101/gad.265629.115

**Published:** 2015-10-01

**Authors:** Madhusoodanan Urulangodi, Marek Sebesta, Demis Menolfi, Barnabas Szakal, Julie Sollier, Alexandra Sisakova, Lumir Krejci, Dana Branzei

**Affiliations:** 1 FIRC (Fondazione Italiana per la Ricerca sul Cancro) Institute of Molecular Oncology (IFOM), 20139 Milan, Italy;; 2 National Centre for Biomolecular Research, Masaryk University, CZ-62500 Brno, Czech Republic;; 3 Department of Biology, Masaryk University, CZ-62500 Brno, Czech Republic;; 4 International Clinical Research Center, Center for Biomolecular and Cellular Engineering, St. Anne's University Hospital Brno, CZ-656 91 Brno, Czech Republic

**Keywords:** DNA damage tolerance, replication, recombination, SUMO, genotoxic stress

## Abstract

Accurate completion of replication relies on the ability of cells to activate error-free recombination-mediated DNA damage bypass at sites of perturbed replication. However, as anti-recombinase activities are also recruited to replication forks, how recombination-mediated damage bypass is enabled at replication stress sites remained puzzling. Here we uncovered that the conserved SUMO-like domain-containing *Saccharomyces cerevisiae* protein Esc2 facilitates recombination-mediated DNA damage tolerance by allowing optimal recruitment of the Rad51 recombinase specifically at sites of perturbed replication. Mechanistically, Esc2 binds stalled replication forks and counteracts the anti-recombinase Srs2 helicase via a two-faceted mechanism involving chromatin recruitment and turnover of Srs2. Importantly, point mutations in the SUMO-like domains of Esc2 that reduce its interaction with Srs2 cause suboptimal levels of Rad51 recruitment at damaged replication forks. In conclusion, our results reveal how recombination-mediated DNA damage tolerance is locally enabled at sites of replication stress and globally prevented at undamaged replicating chromosomes.

## Abstract (precis)

In this study, Urulangodi et al. demonstrate that a SUMO-mediated regulatory mechanism enables recombination-mediated DNA damage tolerance (DDT) specifically at sites of compromised replication forks. By using a combination of genetic, biochemical, and molecular approaches, they identified a SUMO-like domain (SLD)-containing protein, Esc2, that allows optimal recruitment of the Rad51 recombinase at sites of perturbed replication, thus advancing our understanding of DDT and the pathways that support genome integrity.

DNA lesions are perilous to DNA replication and genome integrity. When lesions are encountered during DNA replication, a complex DNA damage response (DDR) is activated to orchestrate local replication activity and damage bypass and adjust various cellular responses (Jackson and Bartek 2009; Branzei and Foiani 2010). A crucial substrate for DDR activation is ssDNA. This is induced by replication stress (Branzei and Foiani 2010) and accumulates at stalled forks as well as behind replication forks reactivated by repriming downstream from the initial stalling lesions (Heller and Marians 2006; Fumasoni et al. 2015). In addition, ssDNA causes activation of conserved DNA damage tolerance (DDT) pathways (Davies et al. 2008; Karras and Jentsch 2010), which promote the filling in of discontinuities and mediate replication in the presence of damaged templates.

Crucial for DDT is the proliferating cell nuclear antigen (PCNA) sliding clamp protein, which, by means of physical interactions with numerous factors, coordinates key DNA transactions during replication, repair, chromatin structure, and assembly (Moldovan et al. 2007). Post-translational modifications of PCNA with ubiquitin and SUMO further modulate its physical interactions and are crucial for controlling the accuracy of replication by affecting the manner in which damage bypass occurs. This is because the two modes of DDT—an error-free mode (known as template switching, which relies on recombination to the newly synthesized strand) and an error-prone mode (largely accountable for mutagenesis and involving specialized translesion synthesis polymerases)—are differentially regulated by PCNA modifications with SUMO and ubiquitin (Branzei 2011; Ulrich and Takahashi 2013).

PCNA ubiquitylation at a conserved lysine (K) residue, K164, is induced by replication conditions associated with fork stalling (Hoege et al. 2002) and has been detected in all eukaryotic species analyzed to date (Ulrich and Takahashi 2013). The ubiquitylation reaction is mediated by a group of conserved ubiquitin conjugation factors that belong to the *RAD6* pathway (Hoege et al. 2002). In this process, the E2 ubiquitin-conjugating enzyme Rad6 acts in complex with the E3 ubiquitin ligase Rad18, a ssDNA-binding protein that recognizes DNA discontinuities induced by fork stalling, to induce PCNA monoubiquitylation. Another E3 ssDNA-binding protein, Rad5 (or its mammalian orthologs, SHPRH and HLTF), together with the heterodimeric E2 complex Ubc13–Mms2 (or UBC13–UEV1 in mammals), can then extend the monoubiquitin modification to K63-linked polyubiquitin chains. The monoubiquitin modification of PCNA favors its interaction with translesion synthesis polymerases and mutagenic bypass (Stelter and Ulrich 2003), while PCNA polyubiquitylation mediates the error-free mode of damage bypass via template switch recombination to the sister chromatid (Papouli et al. 2005; Pfander et al. 2005; Branzei et al. 2008).

In *Saccharomyces cerevisiae*, PCNA association with DNA also causes its modification with SUMO predominantly at K164 and, to a minor extent, K127, resulting in a largely replication-associated modification pattern (Hoege et al. 2002). PCNA SUMOylation at K164 has also been observed in *Xenopus laevis* egg extracts and mammalian cells (Leach and Michael 2005; Gali et al. 2012; Moldovan et al. 2012). Molecular and biochemical investigations indicated that SUMOylation acts together with PCNA polyubiquitylation in template switching (Branzei et al. 2008; Parker and Ulrich 2012). However, precisely how PCNA SUMOylation orchestrates local recombination-mediated tolerance of lesions by error-free template switching while globally preventing other recombination pathways that could endanger genomic stability is not clear at present. Genetic evidence indicates that the dynamic or regulated recruitment of factors such as Srs2 and Elg1, two known readers of PCNA SUMOylation in budding yeast, is important in modulating DDT pathway choice.

Srs2 belongs to the UvrD family of DNA helicases and interacts preferentially with SUMOylated PCNA by means of two adjacent interaction motifs for PCNA and SUMO present at its C terminus (Papouli et al. 2005; Pfander et al. 2005; Armstrong et al. 2012; Kolesar et al. 2012). Biochemically, Srs2 eliminates recombination intermediates by disrupting or preventing the formation of Rad51 presynaptic filaments (Krejci et al. 2003; Veaute et al. 2003; Robert et al. 2006). In higher eukaryotes, the UvrD helicase PARI functionally resembles Srs2 in its anti-recombinase function and preferential binding to SUMOylated PCNA (Moldovan et al. 2012), but whether it is indeed the Srs2 functional ortholog remains unclear. Genetic and molecular data indicated that, following genotoxic stress, Srs2 and PCNA SUMOylation are permissive for error-free Rad5- and Rad51-dependent recombination events while postponing other potentially toxic recombination events for later in the cell cycle (Branzei et al. 2008; Karras et al. 2013).

Budding yeast Elg1 and its homolog, ATAD5, in mammalian cells form an alternate replication factor C-like complex (Bellaoui et al. 2003; Ben-Aroya et al. 2003; Kanellis et al. 2003) that promotes unloading of PCNA during replication (Kubota et al. 2013; Lee et al. 2013). This function is important for genome maintenance but is not essential for replication. *S. cerevisiae* Elg1 interacts preferentially with SUMOylated PCNA via three SUMO-interacting motifs (SIMs) and a PCNA-interacting peptide (PIP)-like motif but also with other SUMOylated proteins (Parnas et al. 2010, 2011). The SUMO interaction function of Elg1 and ATAD5 also contributes to genome stability but appears distinct from its function in PCNA unloading (Parnas et al. 2010, 2011; Kubota et al. 2013). Nevertheless, the conserved SIMs in Elg1 and ATAD5 likely indicate the existence of additional interaction partners with SUMO-like features that have not been yet identified and may modulate DDR and genome stability.

Here we identify the conserved SUMO-like domain (SLD)-containing protein Esc2 as a novel structure-specific DNA-binding factor implicated in local regulation of damage bypass by template switch recombination. Critical for Esc2 function is its binding to stalled replication forks and its subsequent SLD-mediated interaction with the SIMs of Srs2, which subsequently impacts on Esc2's ability to uphold optimal Rad51 binding at sites of compromised replication. The mechanism that we uncovered for Esc2 modulation of Srs2 is two-faceted, involving chromatin recruitment and turnover. We propose that, in the face of genotoxic replication stress, SUMO/SLD-dependent chromatin interactions and proteolytic events are wired to promote local recombination by suppressing the Srs2 helicase, which normally prevents unscheduled recombination at undamaged replicating chromosomes.

## Results

### Esc2 is required for Rad51 recruitment at damaged replication forks

**Figure 1.**
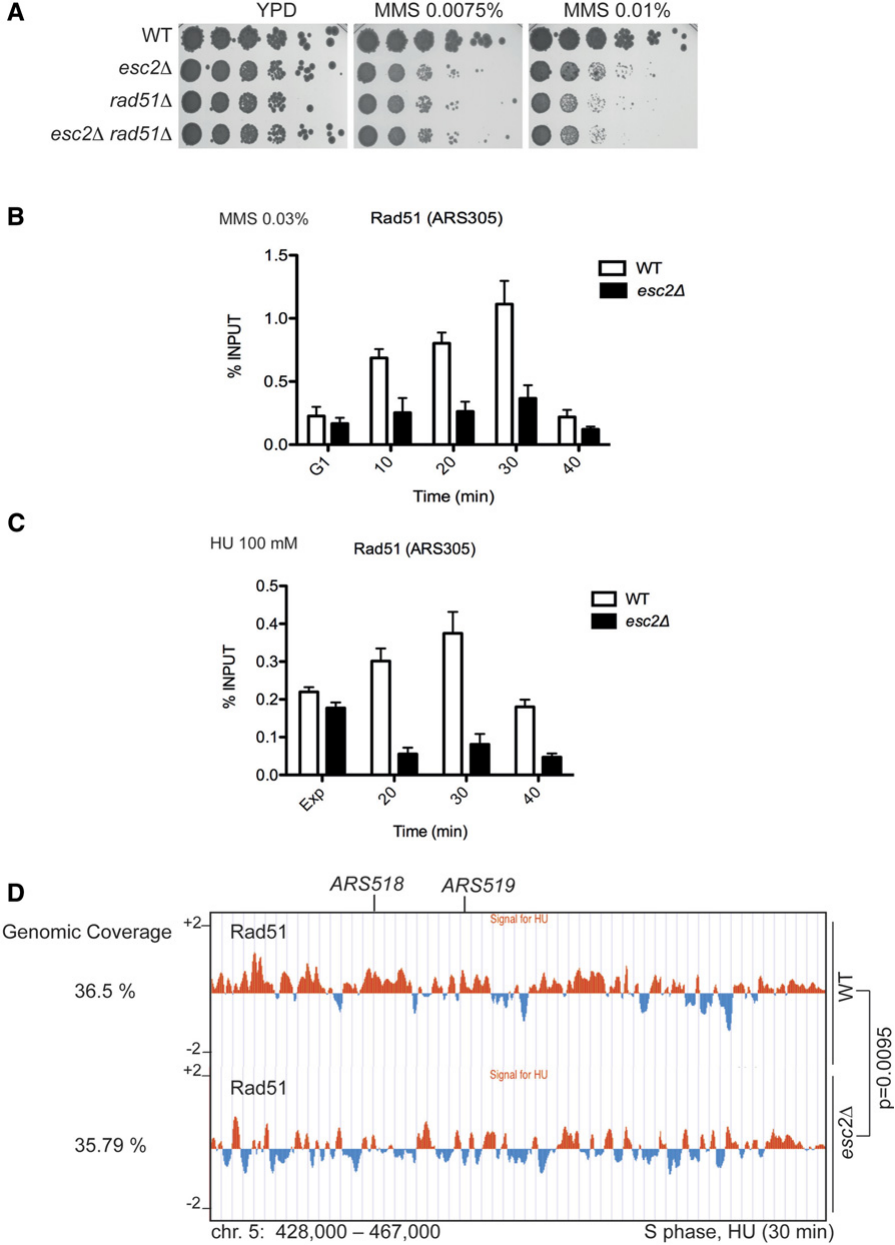
Esc2 facilitates Rad51 recruitment to damaged replication forks. (*A*) The MMS sensitivity of wild-type (WT), *esc2*Δ, *rad51*Δ, and *esc2*Δ *rad51*Δ strains was examined by spot assay. (*B*,*C*) Recruitment of Rad51 to early origins of replication. ChIP-qPCR assay was used to analyze the recruitment of Rad51 to the early origin of replication (*ARS305*) after synchronous release in S phase in the presence of 0.03% MMS (*B*) or 0.1 M HU (*C*) at 28°C. (Exp) Exponentially growing cells; (G1) α-factor-arrested samples. Each ChIP experiment was repeated three times, and each real-time PCR was performed in triplicates. The bar represents the mean value ± standard error of mean (SEM). (*D*) The genome-wide binding pattern of Rad51 in wild-type and *esc2*Δ cells by ChIP-on-chip after synchronous release of cells from G1 arrest in medium containing 0.1 M HU for 30 min. The histogram bars on the *Y*-axis represent the genome browser view of Rad51 binding represented as the average signal ratio in log_2_ scale of loci enriched in the immunoprecipitated fraction along the indicated regions. The *X*-axis shows chromosomal coordinates. The indicated *P*-values relate to the genome-wide overlap between Rad51 clusters in the two strains. Chromosome 5 is shown as a representative example.

Deletion of *ESC2* renders cells sensitive to MMS-induced DNA damage in a manner epistatic with *rad51*Δ, which is deficient in homologous recombination (HR) (Fig. 1A; Mankouri et al. 2009; Sollier et al. 2009). The molecular basis of this repair defect has remained elusive. We asked whether Esc2 absence might affect Rad51 recruitment to damaged or stalled replication forks. To test this, we used chromatin immunoprecipitation (ChIP) combined with quantitative PCR (qPCR) to examine the binding affinity of Rad51 to the early origin of replication, *ARS305*, in wild-type and *esc2*Δ cells following replication fork stalling induced by MMS or hydroxyurea (HU). We observed a marked reduction in Rad51 binding in *esc2*Δ in both experimental conditions (Fig. 1B,C), although no effects on Rad51 protein levels or turnover were observed in *esc2*Δ (data not shown). Importantly, *esc2*Δ cells showed normal S-phase progression (Supplemental Fig. S1A,B). We further examined Rad51 binding at regions containing late/dormant origins that do not contain replication forks at early times during chromosome replication. In this case, we found no difference in Rad51 binding between wild type and *esc2*Δ (Supplemental Fig. S1C,D). Moreover, using the ChIP-on-chip technique, we found significant overlap between genome-wide Rad51 clusters in wild type and *esc2*Δ, with the overall genomic coverage of Rad51 being comparable between the two strains (Fig. 1D). As we did not detect any physical interaction between Esc2 and Rad51 using various approaches (Supplemental Fig. S1E; see below), we reasoned that Esc2's local effect on Rad51 binding is likely indirect. In conclusion, the above results show that Esc2 locally influences Rad51 binding specifically at sites of replication stress, providing a molecular explanation for the observed epistasis between *rad51*Δ and *esc2*Δ mutations with regard to DDT.

### Esc2 interacts with modulators of error-free DDT

**Figure 2.**
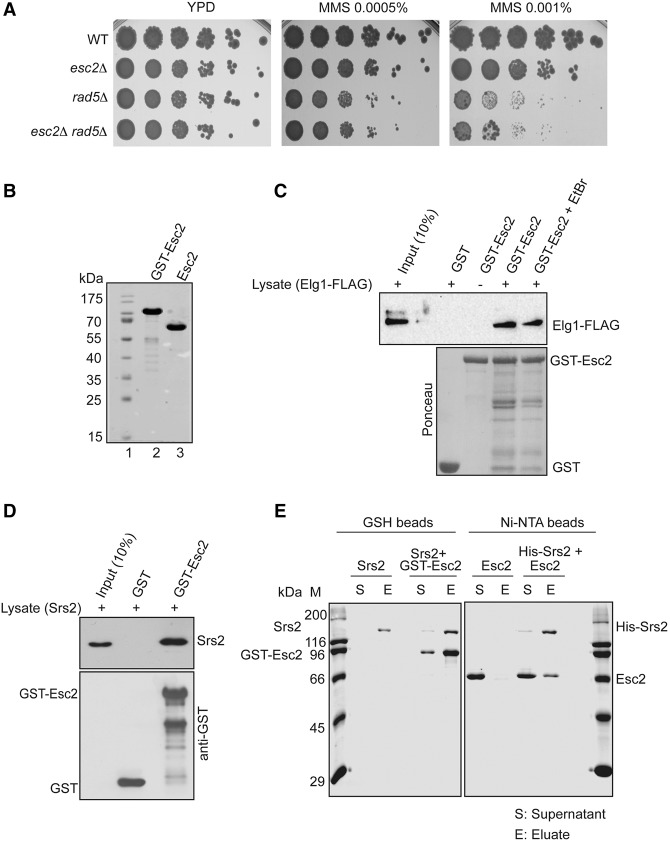
Esc2 interacts physically and functionally with error-free DDT factors and regulators. (*A*) *ESC2* deletion is epistatic with *rad5*Δ for damage sensitivity. The strains of the indicated genotypes were analyzed for MMS sensitivity by spot assay. (*B*) Esc2 protein expressed and purified from *Escherichia coli*. Coomassie-stained samples of purified recombinant proteins used for interaction studies. The positions of molecular weight markers are indicated in lane *1*. (*C*) Recombinant GST-Esc2 protein binds endogenous Elg1-Flag in a GST pull-down assay. The Ponceau S-stained *bottom* panel served as a loading control. Total cell lysates prepared from cells expressing Elg1-Flag were incubated with GST or GST-Esc2 in either the presence or absence of ethidium bromide, and the protein complex formed on the beads was separated on a 10% SDS-PAGE gel. Total cell lysates (10% input) and pull-downs were analyzed by protein blotting using anti-Flag antibody. (*D*) Esc2 physically interacts with Srs2. Same as in *C*, but the total wild-type cell lysates (10% input) and pull-downs were analyzed by protein blotting using anti-Srs2 antibody. (*E*) Esc2 interacts with Srs2 in an in vitro pull-down assay. His-tagged Srs2 (3 μg) was mixed with 3 μg of Esc2 in the presence of Ni-NTA beads or with 3 μg of GST-Esc2 in the presence of glutathione beads. After incubation, the beads were washed and treated with SDS to elute the bound proteins. The supernatants (S) with unbound proteins and the SDS elution (E) fractions were analyzed by SDS-PAGE and visualized by Coomassie staining. Control experiments in which Srs2 was incubated with glutathione beads or in which Esc2 was incubated with Ni-NTA beads are also indicated.

Since *esc2*Δ mutants are not generally defective in recombination (Mankouri et al. 2009; Sollier et al. 2009), in line with our observation that Esc2 does not affect Rad51 recruitment globally (Fig. 1D), we tested the sensitivity of *esc2*Δ in combination with other DDT mutations. We found that *esc2*Δ was epistatic with *rad5*Δ (Fig. 2A), which is deficient in the error-free recombination-mediated damage bypass by template switching (Branzei et al. 2008). As *esc2*Δ did not increase the sensitivity of *rad5Δ rad51*Δ cells (Supplemental Fig. S2A), these results are congruent with a role of Esc2 in the recombination-mediated DDT pathway that depends on both Rad51 and Rad5/PCNA polyubiquitylation activities (Branzei et al. 2008; Choi et al. 2010).

Next, we set out to search for physical interactions between Esc2 and relevant DDT players or modulators. We identified a physical interaction between Esc2 and Srs2 by two-hybrid assay (Supplemental Fig. S2B) but did not observe evidence for interaction between Esc2 and PCNA as assessed by either two-hybrid (Supplemental Fig. S2B) or in vivo pull-down (Supplemental Fig. S2C; see below) assay. For the latter assay, we used recombinant GST-Esc2 fusion protein and cell lysates containing endogenous PCNA. Next, we investigated whether Esc2 interacts with the main regulators and interactors of SUMOylated PCNA; namely, Srs2 (already identified by two-hybrid) (Supplemental Fig. S2B) and Elg1 (Papouli et al. 2005; Pfander et al. 2005; Parnas et al. 2010). To this end, we again carried out pull-down assays using purified recombinant GST-Esc2 (Fig. 2B) or GST alone as a negative control and yeast cell lysates. Elg1-Flag, present in cell lysates, formed a stable complex with GST-Esc2 (Fig. 2C). The Esc2 interaction with Elg1 was not mediated by contaminant DNA potentially present in the lysates, as addition of ethidium bromide did not interfere with the observed binding (Fig. 2C). However, in contrast to *elg1*Δ, the *esc2*Δ mutation did not cause an accumulation of SUMOylated PCNA on chromatin (Supplemental Fig. S2D), indicating that Esc2's function in the Rad5 pathway is diverse from that of Elg1. Using in vivo pull-downs, we also observed an interaction between GST-Esc2 and endogenous Srs2 (Fig. 2D). To further examine whether Esc2 interacts directly with Srs2, we performed in vitro pull-down using purified Srs2 and Esc2 proteins. In these experiments, we incubated GST-Esc2 with His-Srs2 and pulled down the complex on either GSH (Fig. 2E, left panel) or Ni-NTA beads (Fig. 2E, right panel), respectively. In both cases, increased retention of Srs2 and Esc2, respectively, was observed when the beads contained the partner protein. Based on the above results, we conclude that Esc2 physically interacts with the DDT modulators Elg1 and Srs2 and that the interaction with Srs2 is direct.

### The SIMs of Elg1 and Srs2 mediate their interaction with Esc2

Elg1 contains three SIMs and a PIP-like motif in the N-terminal region (Supplemental Fig. S3A). These regions are required for Elg1 interaction with SUMOylated PCNA (Parnas et al. 2010). We asked whether they were also critical for interaction with Esc2. To this end, we mutated the three SIMs and the PIP-like motif of Elg1 individually and in combination and then replaced the *elg1*Δ locus with different *elg1*-modified alleles further tagged C-terminally with Flag. All of the Elg1 variants were stable and were expressed at levels similar to that of wild-type Elg1-Flag (Supplemental Fig. S3B). We then examined the interaction of the corresponding Elg1 variants with GST-Esc2 by in vivo pull-down. Only Elg1 wild-type and Elg1-PIP variants interacted with high efficiency with GST-Esc2, whereas all of the SIM variants analyzed were defective in this interaction (Supplemental Fig. S3C). Interestingly, the *elg1-SIM* alleles partly suppressed *esc2*Δ’s sensitivity to MMS in a manner dependent on the Elg1 PIP motif (Supplemental Fig. S3D). These results suggest that Elg1's functional interaction with Esc2 relies on the ability of Elg1 to bind SUMOylated PCNA.

**Figure 3.**
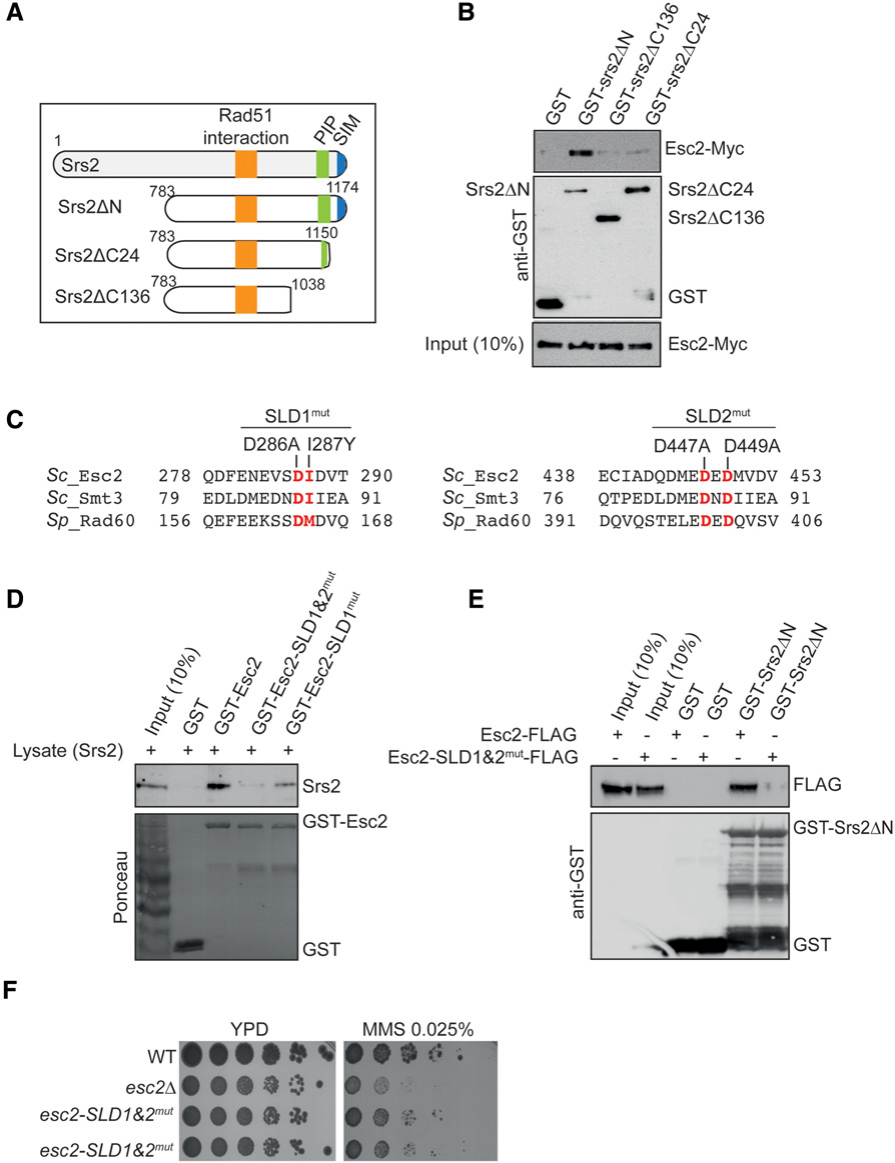
The SIMs of Srs2 mediate physical interaction with the SLDs of Esc2. (*A*) Schematic map of several GST-Srs2 truncations used for pull-down assay. (*B*) GST-Srs2ΔN, GST-Srs2ΔC136, and GST-Srs2ΔC24 pull-downs using total cell lysate from cells expressing Myc-tagged *ESC2* (Esc2-Myc). (*C*) Amino acids sequence alignment of SLD1 and SLD2 of Esc2 with *S. cerevisiae* SUMO (Smt3) and *Schizosaccharomyces pombe* Rad60. The conserved residues chosen for mutagenesis are highlighted in red, and the mutations are annotated. (*D*) Total cell lysates prepared from wild-type cells were incubated with GST or the indicated GST-Esc2 (mutant) proteins. GST pull-down assay was performed as in Figure 2D. The Ponceau S-stained *bottom* panel served as a loading control. (*E*) GST pull-down assay with recombinant GST-Srs2ΔN and cell lysates prepared from Esc2-Flag and Esc2-SLD1&2^mut^-Flag strains. (*F*) The MMS sensitivity of the indicated strains was examined by spot assay.

We next examined the domains of Srs2 that are required for interaction with Esc2. For this purpose, we used an N-terminal-deleted, Srs2ΔN variant (Fig. 3A) lacking the helicase domain but able to bind Rad51 and PCNA with affinities similar to full-length Srs2 (Krejci et al. 2003; Pfander et al. 2005). We constructed two other deletion mutants in Srs2ΔN: Srs2ΔC24 and Srs2ΔC136 (Fig. 3A), which lack the SIM or both the SIM and PIP of Srs2, respectively. We expressed and purified these Srs2 variants as GST fusion proteins (Supplemental Fig. S4A). Next, we performed in vivo pull-down assays using the above-described GST-Srs2ΔN variants and total cell lysates prepared from wild-type cells expressing Esc2-Myc. While Esc2 interacted robustly with GST-Srs2ΔN, deletion of the C-terminal region or even of the last 24 amino acids of Srs2 resulted in a markedly reduced interaction (Fig. 3B). We observed a similar requirement for the C-terminal domain of Srs2 in interacting with Esc2 by yeast two-hybrid experiments (Supplemental Fig. S4B). Moreover, by two-hybrid assay, we found that the C-terminal domains of Srs2 containing the PIP and SIM (fragments 909–1174 and 1036–1174) were also able to interact with full-length Esc2 (Supplemental Fig. S4B). Interestingly, the *srs2ΔC136* variant lacking both the SIM and PIP motifs—but not the *srs2ΔC6* mutant lacking only the SIM—suppressed *esc2*Δ sensitivity to MMS (Supplemental Fig. S4C,D). These results suggest that Srs2 binding to SUMOylated PCNA modulates DDT in *esc2*Δ cells. Based on the above results, we conclude that the Srs2 C-terminal region containing the SIM and PIP is sufficient for Srs2 to engage in interaction with Esc2 and that, within this domain, the SIM motif of Srs2 is critical.

### Contributions of Esc2 SLDs to binding Srs2 and Elg1

The SLDs of Esc2 are the characteristic feature of this protein and represent the most probable interface for its SIM-mediated interaction with Elg1 and Srs2. To examine this, we mutated residues at SLD1 and SLD2 of Esc2 that are conserved in the *S. cerevisiae* SUMO ortholog Smt3 and *Schizosaccharomyces pombe* Rad60. We identified two such residues in SLD1 (D286 and I287) and two in SLD2 (D447 and D449), which we mutated as described in Figure 3C. To test the effect of these mutations on Esc2 interaction with Srs2 and Elg1, we first introduced these mutations in GST-Esc2. The Esc2 recombinant variants were efficiently expressed and purified (Supplemental Fig. S3E) and then tested for their interaction with Srs2 and Elg1 using in vivo pull-down assays. While the introduced SLD mutations did not impair the ability of Esc2 to interact with Elg1 (Supplemental Fig. S3F), the SLD mutations strongly reduced the interaction with Srs2 (Fig. 3D).

Next, we replaced the *esc2*Δ locus with an *esc2-SLD1*&*2*^*mut*^ allele tagged C-terminally with Flag to examine the interaction of this variant with GST-Srs2ΔN. We note that this variant was stable and was expressed at wild-type levels (see below). We also observed in this way that Srs2 interaction with Esc2-SLD1&2^mut^ was strongly reduced in comparison with wild-type Esc2 (Fig. 3E). The *esc2-SLD1*&*2*^*mut*^ also showed sensitivity to MMS but milder than *esc2*Δ (Fig. 3F). We conclude that Srs2 and Esc2 interact via the SIM and SLD interfaces of Srs2 and Esc2, respectively.

### Esc2 binds branched DNA structures in vitro and associates with stalled replication forks in vivo

**Figure 4.**
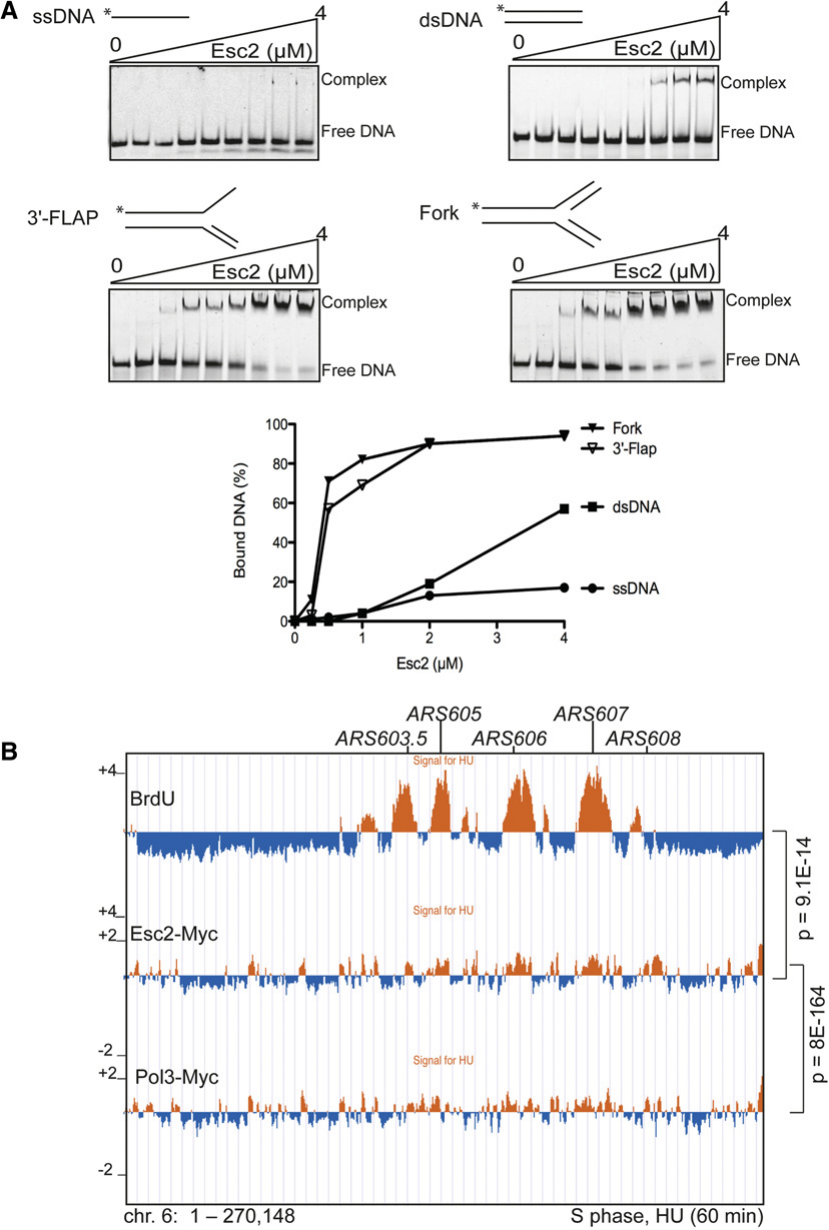
Esc2 is a structure-specific DNA-binding protein that is recruited to stalled replication forks. (*A*) Fluorescently labeled DNA substrates (7 nM) were incubated with increasing concentrations of purified Esc2 for 10 min at 37°C. The gels represent the tested substrates ssDNA, dsDNA, 3′-Flap, and fork. The percentages of bound DNA versus total derived from quantification of individual gels are plotted. (*B*) Genome-wide binding pattern of Esc2-Myc by ChIP-on-chip after synchronous release of cells from G1 arrest in medium containing 0.2 M HU for 60 min. The experiment was performed and analyzed as described in Figure 1D. Control experiments with BrdU and Pol3 are indicated. The overlap between the binding clusters of BrdU and Pol3 with Esc2 ChIP-on-chip is represented. The indicated *P*-values relate to the genome-wide overlap between the considered protein clusters. Chromosome 6 is shown as a representative example.

The observed physical interactions and the DDT roles that we identified for Esc2 prompted us to test whether Esc2 binds directly to different types of DNA structures. Although we did not identify any typical DNA-binding motifs within Esc2 using bioinformatics approaches, we examined the above hypothesis using electrophoretic mobility shift assay (EMSA). For this purpose, we used fluorescently labeled DNA substrates and purified recombinant Esc2 protein without the GST tag (see Fig. 2B). Interestingly, Esc2 bound all of the substrates used in the assay in a concentration-dependent manner, but the binding affinities were markedly different (Fig. 4A). Specifically, Esc2 showed strong preference for branched DNA structures (Flap and fork) and little affinity for ssDNA (Fig. 4A), making *S. cerevisiae* Esc2 a structure-selective DNA-binding protein.

Next, we examined whether Esc2 is enriched at genomic regions containing stalled replication forks in vivo. To this end, we used a ChIP-on-chip approach to identify Esc2 chromatin positions when cells were synchronized in S phase by treatment with HU (Fig. 4B). We found that Esc2 associates with 67.4% of early *ARS* regions that fire under our experimental conditions, with the enrichment of Esc2 at early *ARS* regions being statistically highly significant (*P*-value of 1.1 × 10^−6^). Moreover, the genome-wide clusters of Esc2 showed statistically significant overlap with clusters for both BrdU and the DNA polymerase δ catalytic subunit Pol3 (Fig. 4B). Thus, we conclude that Esc2 binds to regions of perturbed replication in vivo, where it facilitates recombination-mediated DDT.

### Distinct Esc2 domains implicated in binding replication forks and Srs2

**Figure 5.**
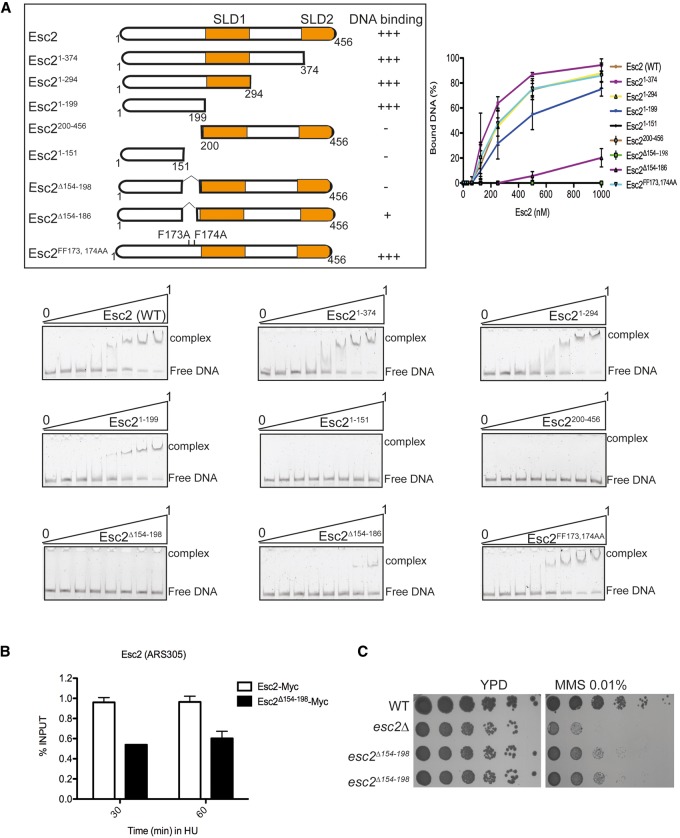
Mapping of the Esc2 DNA-binding domain. (*A*) Schematic map of various Esc2 truncation variants used for EMSA assay. The assay was performed as in Figure 4A using 7 nM fluorescently labeled fork substrate, increasing concentrations of purified Esc2, and various truncations (0–1000 nM). The fluorescent DNA species were visualized and quantified using Fuji FLA 9000 imager with Multi-Gauge software (Fuji). (*B*) Recruitment of DNA-binding domain mutant Esc2 (Esc2^Δ154–198^-Myc) to the early origin of replication (*ARS305*) by ChIP-qPCR. Samples were collected at 30 and 60 min after synchronous release in S phase in the presence of 0.1 M HU at 28°C. (*C*) The MMS sensitivity of the indicated strains was examined by spot assay.

With the aim of mapping the Esc2 domain implicated in binding stalled replication forks, we next constructed several Esc2 truncation variants (Fig. 5A). These recombinant proteins were expressed and purified from *Escherichia coli* and tested for their ability to bind a fork substrate by EMSA (see Fig. 4A). Esc2 variants that partly or completely missed the SLDs (1–199 amino acids) were highly proficient in binding, whereas an Esc2 peptide containing the SLDs did not show binding activity (Fig. 5A). When we further truncated the N-terminal part of Esc2 to the first 151 amino acids, the DNA-binding activity was completely lost, indicating that the Esc2 domain spanning between 151 and 199 amino acids comprised this binding activity. To further test this contention, we established and purified two full-length Esc2 variants carrying internal truncations within this domain and a full-length variant mutated in two proximal phenylalanines (FF173 and 174AA). The Esc2 variant carrying the 154- to 198-amino-acid internal truncation (Esc2^Δ154–198^) was highly defective in binding, whereas the other two were still proficient to different degrees (Fig. 5A). Importantly, the Esc2^Δ154–198^ variant was still able to bind Srs2, as assessed by in vitro pull-down experiments (Supplemental Fig. S5A) and consistent with the notion that the SLDs of Esc2 provide the interface for interaction with Srs2 (Fig. 3D,E). Thus, Esc2^Δ154–198^ is specifically defective in binding structured DNA.

To next test the effect of this internal truncation in vivo, we replaced the *esc2*Δ locus with an *esc2*^Δ*154–198*^ allele. Importantly, the Esc2^Δ154–198^ variant was highly defective in binding to stalled replication forks, as assessed by ChIP-qPCR at an early origin of replication (*ARS305*) (Fig. 5B), but bound as efficiently as wild-type Esc2 to a late origin of replication (Supplemental Fig. S5B). Moreover, *esc2*^Δ*154–198*^ cells were sensitive to MMS, with this sensitivity being higher than the one of *esc2-SLD1*&*2*^*mut*^ (see Fig. 3F) but less pronounced than the one of *esc2*Δ (Fig. 5C). In contrast to the Esc2^Δ154–198^ variant, Esc2-SLD1&2^mut^ was only mildly deficient in binding stalled forks (Supplemental Fig. S5C,D), and both the Esc2^Δ154–198^ and Esc2-SLD1&2^mut^ variants were stable and expressed at wild-type levels (Supplemental Fig. S5E). Notably, Esc2^Δ154–198^ was still proficient in binding chromatin genome-wide, as assessed by ChIP-on-chip, and had only partly reduced genomic coverage (Supplemental Fig. S5F). Together, these results reveal that the ability of Esc2 to directly bind to stalled replication forks is crucial for DDT.

### Esc2 facilitates robust Elg1 binding to stalled replication forks and down-regulates Srs2

**Figure 6.**
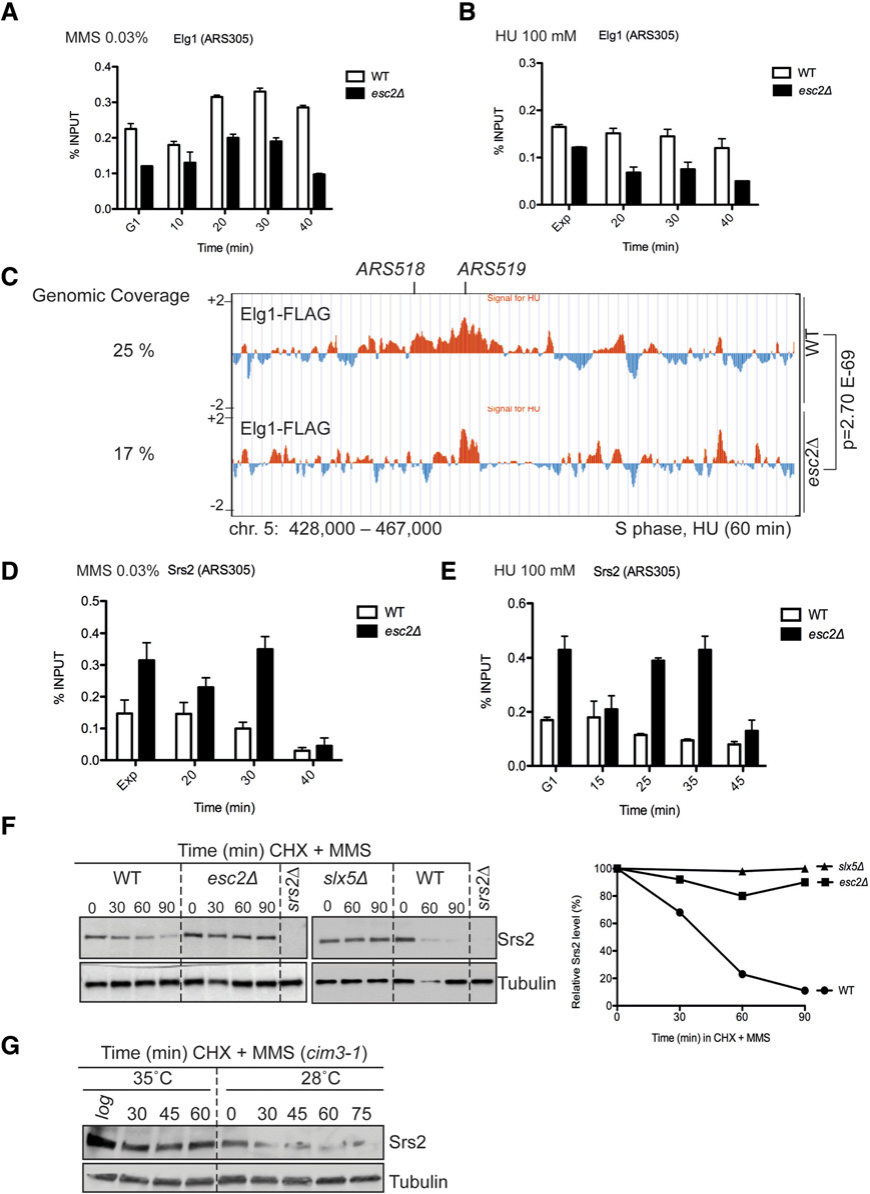
Esc2 differentially influences Elg1 and Srs2 recruitment at damaged replication forks. (*A*,*B*) ChIP-qPCR assays to analyze the recruitment of Elg1-Flag to the early origin of replication (*ARS305*) following synchronous release of G1 cells in medium containing 0.03% MMS (*A*) or 0.1 M HU (*B*) at 28°C. (*C*) Genome-wide binding pattern of Elg1-Flag in wild-type and *esc2*Δ cells by ChIP-on-chip. The indicated *P*-values relate to the genome-wide overlap between Elg1 clusters in the two strains. (*D*,*E*) ChIP-qPCR assay to measure the recruitment of Srs2 to *ARS305* in the presence 0.03% MMS (*D*) or 0.1 M HU (*E*). (*F*) Esc2 and Slx5–Slx8 mediate turnover of the Srs2 helicase. The stability of the endogenous Srs2 protein analyzed by cycloheximide (CHX) chase experiments. Wild-type, *esc2*Δ, and *slx5*Δ cells were arrested in G1 and released into YPD medium containing 0.03% MMS and 50 μg/mL CHX. Protein samples were collected at the indicated time points and analyzed using an anti-Srs2 antibody, the specificity of which was confirmed in each experiment using the *srs2*Δ strain as a control. Tubulin staining served as a loading control. The percentage values of Srs2 versus tubulin, obtained after quantification of band intensities, are plotted. (*G*) Srs2 turnover is proteasome-dependent. Srs2 protein levels are stabilized in the proteasome-deficient mutant *cim3-1* at the nonpermissive temperature of 35°C.

Since both Esc2 and Elg1 associate with chromatin and interact with each other (Fig. 2C), we asked whether Esc2 affects Elg1 association to chromatin in S phase. To this end, we first analyzed by ChIP-qPCR the binding of Elg1 at an early origin (*ARS305*) during replication in the presence of either MMS or HU. The binding of Elg1 was significantly reduced at this early origin of replication in *esc2*Δ cells compared with wild type following either MMS or HU treatment (Fig. 6A,B). Notably, no effects on Elg1 levels or turnover were observed in *esc2*Δ (data not shown). We further compared the effects of *esc2*Δ on Elg1 chromatin binding with those of the SIM mutations or the SIM and PIP mutations of Elg1. The effects of *esc2*Δ were modest in comparison with the Elg1 SIM mutations, and the combination of SIM and PIP mutations in Elg1 very strongly reduced its association to *ARS305* (Supplemental Fig. S6A). Notably, the observed effects on Elg1 binding were limited to active regions of replication and were not observed at late origins of replication (Supplemental Fig. S6B). Moreover, when we analyzed the genome-wide Elg1 clusters obtained by ChIP-on-chip, we found statistically significant overlap with the ones of BrdU and the polymerase δ subunit Pol3 (Supplemental Fig. S6C). Together, the results indicate that Elg1 is recruited to stalled forks primarily via its SIM-mediated interaction with SUMOylated PCNA, but Esc2 contributes to stabilizing or prolonging Elg1 association to stalled forks.

To test further whether Esc2 influences Elg1 clusters genome-wide, we performed ChIP-on-chip of Elg1-Flag in the presence of HU. Also in this case, the absence of Esc2 led to a reduction in the Elg1 peaks, particularly visible at the early origins of replication, but without drastically affecting the genome-wide clusters of Elg1 (Fig. 6C). Specifically, 78% of the early origins of replication showed qualitatively reduced Elg1 binding in *esc2*Δ as assessed by ChIP-on-chip, a phenotype confirmed quantitatively by ChIP-qPCR (Fig. 6B). Based on these results, we conclude that Esc2 upholds robust association of Elg1 to regions of replication stress.

Reduction in Elg1 binding at stalled forks in *esc2*Δ may lead to increased local concentration of SUMOylated PCNA (Parnas et al. 2010) and, consequently, its binding partner, the anti-recombinase Srs2 (Papouli et al. 2005; Pfander et al. 2005). Indeed, when we analyzed Srs2 binding at damaged or stalled forks by ChIP-qPCR, we found this to be increased in *esc2*Δ compared with wild type (Fig. 6D,E). This effect was again specific to sites of active replication and was not observed at late ARS regions (Supplemental Fig. S6D,E). We further considered that Esc2 may act directly in tuning down the Srs2 anti-recombinase activity but did not observe any effect on the ability of Srs2 to disrupt D loops by disassembling Rad51 filaments using D-loop in vitro assays (Krejci et al. 2003; data not shown). In conclusion, the altered patterns of Elg1 and Srs2 chromatin binding in *esc2*Δ provide a mechanistic explanation for the observed reduced Rad51 binding specifically at sites of perturbed replication in this mutant.

### Esc2 and Slx5–Slx8 promote Srs2 turnover

The increased retention of the Srs2 anti-recombinase at damaged forks in *esc2*Δ cells may be solely the result of its increased local association with SUMOylated PCNA. We note that, in contrast to *elg1*Δ, *esc2*Δ mutation did not cause a global increase in PCNA SUMOylation (Supplemental Fig. S2D). These results once again indicate that the effects of Esc2 on Elg1, PCNA SUMOylation, Srs2, and Rad51 are not global but localized. Next, we asked whether the increased Srs2 association to damaged forks in *esc2*Δ is compounded by elevated Srs2 levels, possibly because of Srs2 being abnormally stabilized. To address this, we added cycloheximide (CHX) to the wild-type and *esc2*Δ yeast cultures to inhibit new protein synthesis and monitored the turnover of endogenous Srs2 at different time points upon synchronous release of G1-arrested cells in medium containing MMS. Srs2 levels decreased gradually in wild-type cells after CHX addition, whereas, on the contrary, Srs2 was stabilized in *esc2*Δ (Fig. 6F). Thus, along with cell cycle-dependent transcriptional regulation (Heude et al. 1995), our findings identify Esc2-mediated turnover as a new mechanism controlling Srs2 levels.

The SUMO targeted ubiquitin ligase (STUbL) Slx5–Slx8 complex plays a role in genome stability by controlling the turnover of SUMOylated factors in response to DNA damage (Sriramachandran and Dohmen 2014). As Esc2 and its *Schizosaccharomyces pombe* ortholog, Rad60, genetically and physically interact with Slx5–Slx8 (Prudden et al. 2007; Sollier et al. 2009) and as Srs2 is SUMOylated (Saponaro et al. 2010; Kolesar et al. 2012), we examined whether Srs2 degradation is also mediated by Slx5–Slx8. We found that Srs2 protein levels were stabilized following genotoxic stress in the absence of Slx5, similar to what we observed in *esc2*Δ (Fig. 6F). Comparable results were obtained in *slx8*Δ cells (data not shown). Moreover, using in vivo pull-down assays, we found that both Esc2 and Srs2 interact with Slx5 (Supplemental Fig. S7A,B). Slx5 contains multiple SIMs that mediate its role in protein turnover (Sriramachandran and Dohmen 2014). We found that the SLD1&2 mutations in Esc2 strongly reduced its interaction with Slx5 (Supplemental Fig. S7A), but an Srs2 C-terminal truncation that was defective in interacting with Esc2 (Fig. 3B) was still proficient in binding Slx5 (Supplemental Fig. S7B).

The Slx5–Slx8 complex generally promotes degradation of SUMOylated targets via a proteasome-dependent pathway (Sriramachandran and Dohmen 2014). Indeed, using a temperature-sensitive proteasome mutant, *cim3-1*, we found that Srs2 turnover was dependent on proteasome function (Fig. 6G). Additionally, inhibition of proteasome activity with MG132 similarly led to stable Srs2 levels (data not shown).

We next addressed whether defects in Slx5-mediated Srs2 turnover will cause higher levels of Srs2 and decreased levels of Rad51 at regions of perturbed replication. This was indeed the case, as revealed by ChIP-qPCR of Srs2 and Rad51 binding at early origins of replication in *slx5*Δ cells (Supplemental Fig. S7C,D). Also in this case, no effects of *slx5*Δ on Srs2 and Rad51 binding at a late origin of replication were observed (Supplemental Fig. S7E,F). Taken together, these results indicate that Esc2 and Slx5–Slx8 jointly mediate proteasome-dependent Srs2 degradation. Moreover, this pathway acts in conjunction with Elg1-mediated regulation of chromatin-associated PCNA to limit the levels of the anti-recombinase Srs2 at sites of replication stress.

### Esc2 functions enabling optimal Rad51 recruitment to stalled replication forks

**Figure 7.**
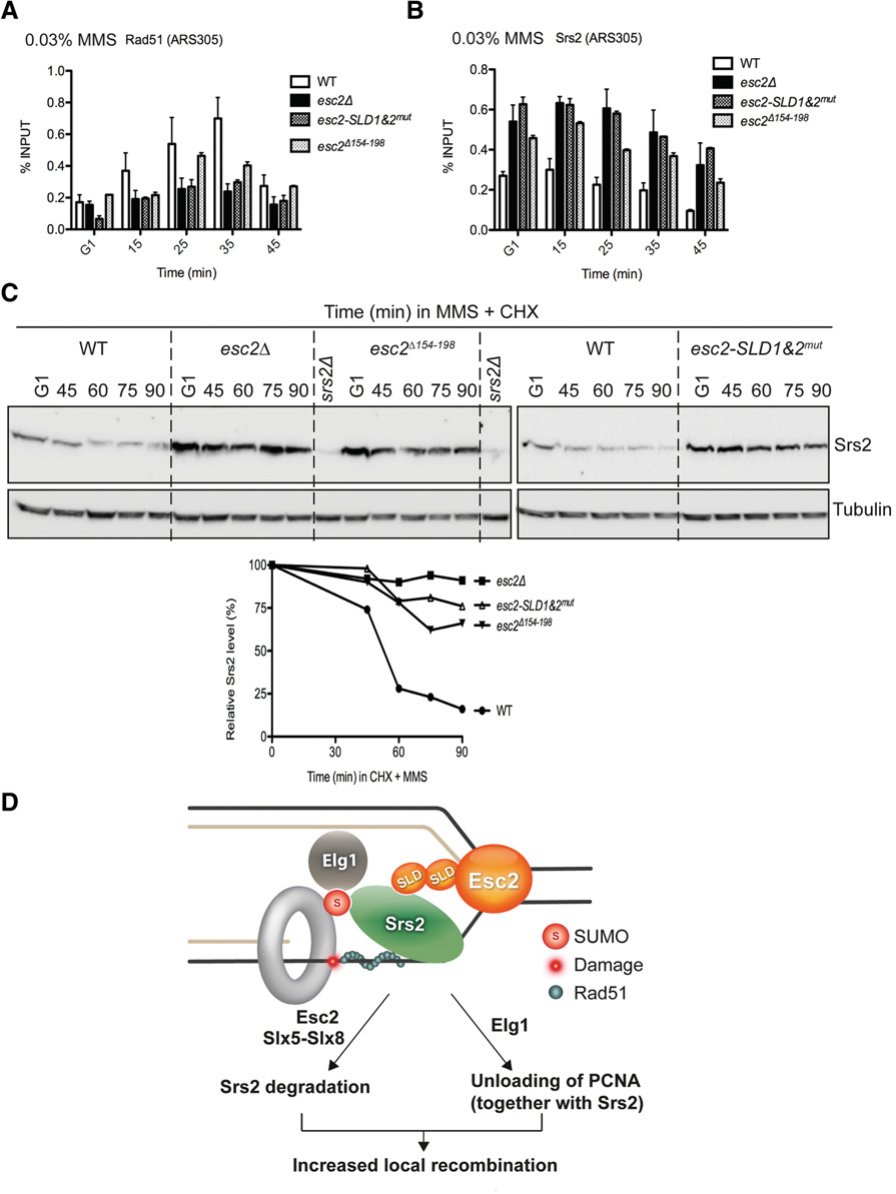
The contribution of Esc2's SLDs and stalled fork-binding properties to Srs2 and Rad51 recruitment at stalled forks. (*A*,*B*) ChIP-qPCR assays to analyze the recruitment of Srs2 and Rad51 to the early origin of replication (*ARS305*) following synchronous release of G1-arrested cells in medium containing 0.03% MMS at 28°C in wild type, *esc2*Δ, *esc2-SLD1*&*2*^*mut*^, and *esc2*^Δ*154–198*^. (*C*) The stability of endogenous Srs2 protein analyzed by CHX chase experiments in wild-type, *esc2*Δ, *esc2-SLD1*&*2*^*mut*^, and *esc2*^Δ*154–198*^ strains. The percentage values of Srs2 versus tubulin, obtained after quantification of band intensities, are plotted. (*D*) A hypothetical model for the role of Esc2 in promoting local recombination. Structure-specific DNA-binding SLD protein Esc2 bound at sites of stalled replication channels Srs2 for Slx5–Slx8-mediated proteasome-dependent degradation and promotes Elg1 association to damaged forks. Increased Srs2 turnover and Elg1-regulated local unloading of the Srs2 chromatin recruiter SUMOylated PCNA locally limit Srs2 helicase levels, facilitating recombination-mediated damage bypass via template switching at sites of perturbed replication.

The two features of Esc2 that we uncovered—that is, its ability to bind replication-related DNA structures and its direct interaction with Srs2—could modulate its role in enabling recombination at stalled forks. To test this, we used the *esc2-SLD1*&*2*^*mut*^ allele (encoding an Esc2 variant defective in the Esc2–Srs2 interaction but proficient in binding stalled forks) (Fig. 3D,E; Supplemental Fig. S5C) and the *esc2*^Δ*154-198*^ allele (encoding a protein defective in binding stalled forks but proficient in interaction with Srs2) (Fig. 5A,B; Supplemental Fig. S5A,C). Notably, both alleles caused a reduction in Rad51 recruitment as well as an enrichment of Srs2 at sites of stalled replication forks (Fig. 7A,B) but not at late origins that did not replicate under our experimental conditions (Supplemental Fig. S8A,B), with *esc2-SLD1*&*2*^*mut*^ being slightly more severe than *esc2*^Δ*154–198*^ and qualitatively identical with *esc2*Δ for the analyzed phenotypes. Moreover, both *esc2* alleles were defective in Srs2 turnover (Fig. 7C). Based on these results, we conclude that Esc2 binds stalled fork DNA structures and subsequently enables local recombination by curbing down the levels of the anti-recombinase Srs2 at sites of perturbed replication.

## Discussion

Two main modes of DDT are present in all eukaryotic organisms and are governed by SUMO and ubiquitin modifications of PCNA (Bergink and Jentsch 2009). One DDT mode, facilitated by PCNA modification with monoubiquitin, uses translesion synthesis polymerases capable of replicating across DNA lesions but also introducing mutations. The other DDT mode is mediated by recombination and involves a switch from the damaged template to a homologous one, usually the sister chromatid (therefore, template switching). Crucial for ensuring correct timing of template switching is the SUMOylation of PCNA (Branzei et al. 2008; Karras et al. 2013). Mechanistically, PCNA SUMOylation prevents unwanted and potentially toxic recombination by recruiting the Srs2 anti-recombinase (Papouli et al. 2005; Pfander et al. 2005; Motegi et al. 2006), but how it still enables template switching, which also depends on recombination activities, remains puzzling. Thus, dedicated mechanisms are expected to exist to locally and temporally facilitate template switching. To date, these mechanisms have remained elusive.

Here we uncovered a SUMO-mediated regulatory mechanism relying on interactions between factors containing SLDs and SIMs that locally coordinate recombination-mediated DDT in conjunction with PCNA SUMOylation. Our new results revealed a two-faceted mechanism involving chromatin recruitment and turnover by which SLD/SIM interactions mediate a decrease in the amount of the Srs2 anti-recombinase specifically at sites of replication stress to enable local recombination (Fig. 7D). Failure to bypass replication-blocking lesions is likely to result in an increased formation of double-strand breaks (DSBs), the repair of which can result in genome aberrations such as gross chromosomal rearrangements and duplications. Thus, our results also offer a molecular rationale for the replication-associated genome aberrations characteristic of mutants defective in template switching (Putnam et al. 2010) and reveal why mutations in Esc2 and Slx5 resemble each other and template switch mutants with regard to such genomic aberrations (Fig. 7D; Albuquerque et al. 2013).

Crucial for recombination reactions is the ability of cells to form and regulate the fate of Rad51 filaments required for strand invasion and extension. Srs2 is a translocase that prevents HR by dismantling Rad51 filaments (Krejci et al. 2003; Veaute et al. 2003) and regulates the extent of DNA repair synthesis in a SUMO–PCNA-dependent manner (Burkovics et al. 2013). Here we uncovered that, in response to damage during replication, SUMO-mediated interactions and proteasome-dependent turnover intersect to promote local down-regulation of Srs2 and facilitate recombination-mediated DDT (Fig. 7D). STUbLs are known to mediate proteasome degradation of SUMOylated substrates (Sriramachandran and Dohmen 2014), and Srs2 is itself SUMOylated (Saponaro et al. 2010). Importantly, here we identified that Esc2 functions upstream of Slx5–Slx8-mediated action as a crucial regulator of Srs2 turnover (Fig. 6F). This function requires robust binding of Esc2 to stalled forks and its subsequent SLD-mediated interaction (Fig. 7C). We propose that Esc2 SLDs act as a platform to recruit Slx5–Slx8 to its substrates at stalled forks and possibly in other chromosomal contexts in which Esc2 functions are important. In this way, STUbL substrates may not necessarily need to be SUMOylated in order to be degraded, as Esc2 could recruit Slx5–Slx8 via its SLDs to the substrates. Notably, adding to the previously identified pathway of Srs2 recruitment to chromatin by SUMOylated PCNA (Papouli et al. 2005; Pfander et al. 2005), our findings identified a new mechanism (orchestrated by the SLD-containing protein Esc2) that acts to locally limit Srs2 levels. Thus, two SUMO-mediated pathways act in distinct ways and cross-talk to regulate local responses to DNA damage.

Genetically, Esc2 function in DDT is performed in the context of error-free recombination by template switching. Mechanistically, we found that Esc2 preferentially binds replication fork-like structures, and it is in this environment that Esc2 engages via its SLDs in regulatory interactions with SIM-containing replisome-associated proteins (Fig. 7D). The results reveal that, following its association with stalled forks, Esc2 interacts with Elg1, Srs2, and Slx5 (also recruited to sites of perturbed replication), causing Srs2 displacement and turnover by the mechanisms discussed above (Fig. 7D). This in turn facilitates local Rad51 filament formation and template switching at damaged sites or stalled replication forks.

Previous work identified Esc2 as important for preventing the accumulation of replication-associated recombination intermediates following genotoxic stress, as revealed by the persistence of X-shaped recombination structures visualized by two-dimensional (2D) gel electrophoresis in *esc2*Δ cells (Mankouri et al. 2009; Sollier et al. 2009). To date, this phenotype was observed in mutants affecting Sgs1/BLM-Top3, the structural maintenance of chromosomes complex Smc5–6, and Esc2 (Liberi et al. 2005; Branzei et al. 2006; Mankouri et al. 2009; Sollier et al. 2009; Choi et al. 2010). While *sgs1*Δ mutants appear to be defective in resolving specific types of recombination intermediates mediating template switching (Giannattasio et al. 2014), to what extent *esc2*Δ and *smc5/6* alleles are similar to *sgs1*Δ in this regard is not known. Here we found that *esc2-SLD1*&*2*^*mut*^ and *esc2*^Δ*154–198*^ alleles phenotypically resemble *esc2*Δ with respect to the X-molecule accumulation phenotype (Supplemental Fig. S9). This phenotype may be due to impaired resolution of the recombination intermediates, and, in this case, our results imply that Esc2 function would involve the DNA structure-selective binding activity of Esc2 and interaction via its SLDs with factors critical for resolution. Alternatively, the defects in enabling error-free recombination-mediated DDT, common for all of the analyzed *esc2* alleles (Fig. 7A–C), may cause increased fork breakage and subsequently lead to deleterious recombination events manifested via the formation of X-shaped intermediates. Future studies will be required to elucidate the complex roles of multitasking and adaptor-like proteins, such as Esc2, in DDT and genome maintenance pathways.

In conclusion, our study provides mechanistic insights into how a conserved SLD protein enables versatile and fine-tuned local responses to DNA damage during replication. The results also highlight a cross-talk between SUMO-mediated post-translational modification and proteolytic turnover, underscoring the intricate control that is imposed during replication on recombination activities in order to promote and maintain genome stability.

## Materials and methods

### Yeast strains and plasmids

Most yeast strains used in this study were derivatives from W303. All genotypes are listed in Supplemental Table S1. The constructs used for protein expression and two-hybrid assays are described in the Supplemental Material.

### Drug sensitivity assay

For qualitative analysis of drug sensitivity, cells from overnight cultures were counted and diluted before being spotted on YPD plates containing the indicated concentrations of MMS and incubated for 2–3 d at 28°C.

### Protein techniques and interaction assays

For GST pull-downs, GST-Esc2 (wild type and point mutants) and GST-Srs2 as well as various GST-tagged truncations were expressed and purified as described in the Supplemental Material. Yeast native extracts for pull-down assays were prepared by using liquid nitrogen, largely as described in Sollier et al. (2009) and detailed in the Supplementa Information. The in vitro pull-down assays were performed largely as in Colavito et al. (2009) and as detailed in the Supplemental Material. EMSAs were conducted as in Marini and Krejci (2012) and as detailed in the Supplemental Material. Yeast two-hybrid assays were performed as described previously (Sollier et al. 2009).

### Antibodies

As antibodies, anti-Flag M2 (Sigma), anti-Rad51 (y-180, Santa Cruz Biotechnology), anti-HA (ab9110, Abcam), anti-Srs2 (yC-18, Santa Cruz Biotechnology), anti-PCNA (ab70472, Abcam), anti-Myc (9E10, sc-40, Santa Cruz Biotechnology), anti-V5/PK (ABD, Serotec), anti-BrdU (MBI-11-13, MBL), and α-tubulin (Sigma) were used.

### ChIP experiments

For ChIP experiments, cells were arrested in G1 with α factor and released at 28°C in medium containing 0.03% MMS or 0.1 M HU. Samples were collected at the indicated time points and fixed with 1% formaldehyde for 15 min. Immunoprecipitation was performed with anti-BrdU, anti-Flag M2, anti-Rad51, anti-HA, anti-Myc, or anti-Srs2 antibody using Dynabeads Protein A (Invitrogen) magnetic beads. Each ChIP experiment was repeated at least three times, and each real-time PCR was performed in triplicates using a Roche LightCycler 480 system and ARS305F–ARS305R primers (Supplemental Table S2) for *ARS305* (early origin) or trs31F–trs31R primers (Supplemental Table S2) for a late origin of replication (*ARS440*). The QuantiFast kit (SYBR Green PCR kit, Qiagen) was used according to the manufacturer's recommendations. The normalization for each data set was performed by subtracting the background signal obtained from immunoprecipitation using the strain *rad51*Δ, *elg1*Δ, or *srs2*Δ as appropriate and indicated. The results were analyzed with absolute quantification/second derivative maximum (Roche LightCycler 480) and the 2^−ΔC(T)^ method as previously described (Livak and Schmittgen 2001).

### ChIP-on-chip

The ChIP-on-chip experiments and statistical analysis of genome-wide clusters were performed as described previously (Bermejo et al. 2009). Two-hundred milliliters of wild-type (Elg1-Flag) and *esc2*Δ (Elg1-Flag) were arrested in G1 (α factor) and then released into medium containing 0.1 M HU for 30 min for Rad51 or 60 min for Elg1. Samples were fixed with 1% formaldehyde for 15 min. For the Esc2-Myc ChIP-on-chip experiment, G1-arrested cells were released in medium containing 0.2 M HU for 60 min and fixed with 1% formaldehyde for 120 min. Immunoprecipitation was performed with anti-BrdU, anti-Flag M2, anti-Myc, or anti-Rad51 antibody using Dynabeads Protein A (Invitrogen). The experiments were performed twice with reproducible results. The microarray data are available online in Gene Expression Omnibus under series number GSE65701 (http://www.ncbi.nlm.nih.gov/geo/query/acc.cgi?token=qngtgiqkdvmdxkr&acc=GSE65701).

### FACS and 2D gel analysis

FACS and 2D gel analysis were performed as previously described (Szakal and Branzei 2013).

## Supplementary Material

Supplemental Material
