# Supplementary material for: Local regulation of the Srs2 helicase by the SUMO-like domain protein Esc2 promotes recombination at sites of stalled replication
Source: Genes Dev. 2015 Oct 1;29(19):2067–80. doi: 10.1101/gad.265629.115 (PMC4604347; doi:10.1101/gad.265629.115)
Supplement: Supplemental Material [file supp_29_19_2067__index.html]

Supplemental Material 

# Local regulation of the Srs2 helicase by the SUMO-like domain protein Esc2 promotes recombination at sites of stalled replication

## Supplemental Material

**Files in this Data Supplement:**

- Supp Material.docx
