# Supplementary material for: Local regulation of the Srs2 helicase by the SUMO-like domain protein Esc2 promotes recombination at sites of stalled replication
Source: Genes Dev. 2015 Oct 1;29(19):2067–80. doi: 10.1101/gad.265629.115 (PMC4604347; doi:10.1101/gad.265629.115)
Supplement: Supplemental Material [file supp_29.19.2067_SuppMaterial.docx]

**Supplemental Information**

**Local regulation of the Srs2 helicase by the SUMO-like domain protein Esc2 promotes recombination at sites of stalled replication**

Madhusoodanan Urulangodi, Marek Sebesta, Demis Menolfi, Barnabas Szakal, Julie Sollier, Alexandra Sisakova, Lumir Krejci, and Dana Branzei

**Supplemental Figures and figure legends**


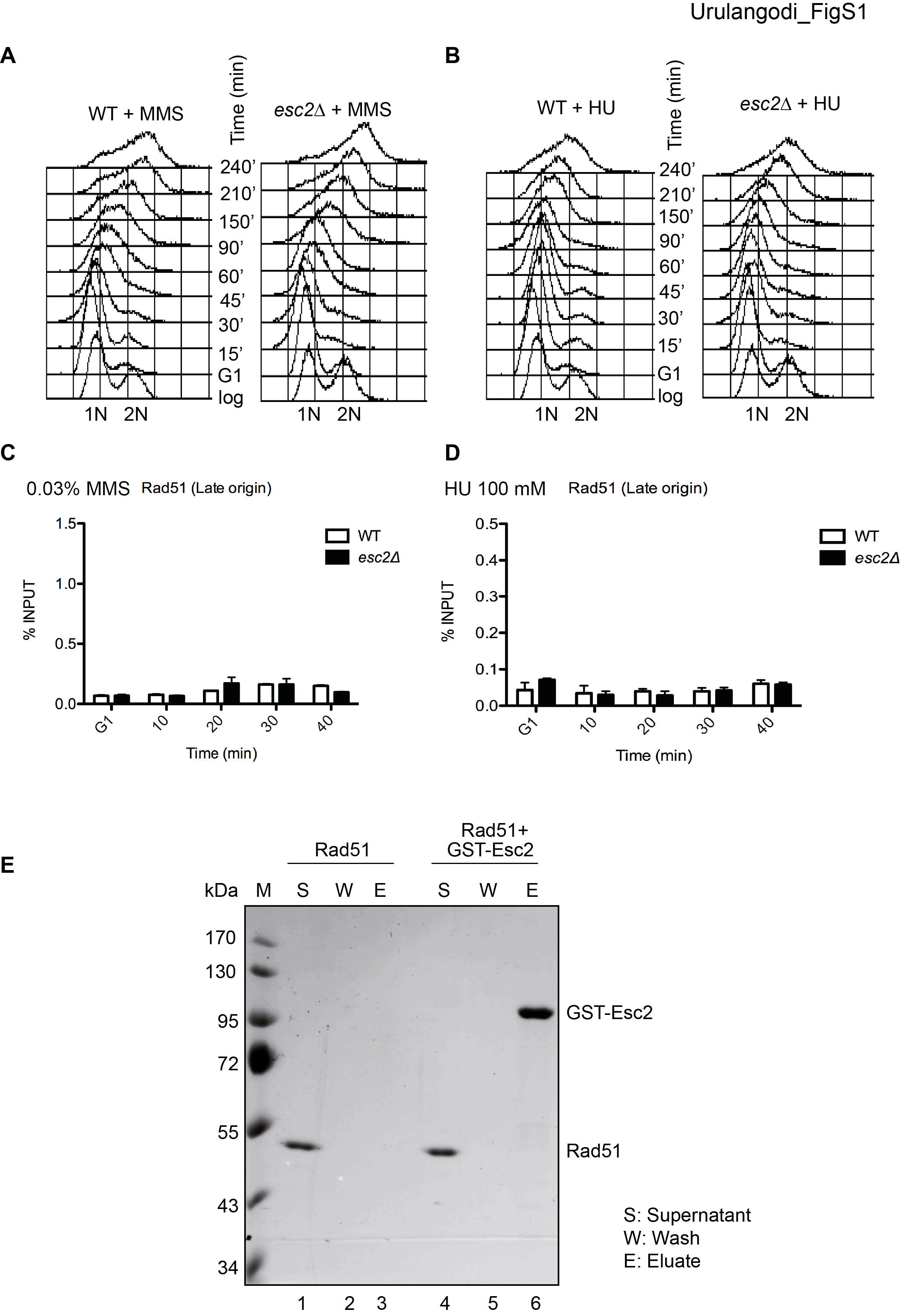


**Figure S1** Esc2 indirectly and only locally affects Rad51 recruitment to chromatin. *(A-B)* FACS plots showing S phase progression of WT and *esc2Δ* cells synchronously released from G1 arrest in media containing MMS 0.033% or HU 0.2 M, respectively. *(C-D)* Rad51 binding at late/dormant origins. ChIP-qPCR assay was performed to analyze the recruitment of Rad51 to a dormant/late origin (ARS440) during replication in the presence of MMS 0.033% or 0.1 M HU. The samples were the same as in Figure 1C. *(D)* Physical interaction between Esc2 and Rad51 was not observed in an *in vitro* pull down assay. GST-Esc2 (5 μg) was mixed with Rad51 (5 μg) in the presence of glutathione beads (lanes 4-6). After incubation, the beads were washed and treated with SDS to elute bound proteins. The supernatants (S) with unbound proteins; the wash (W) and the SDS elution (E) fractions were analyzed by SDS-PAGE and visualized by Coomassie staining. *Lanes 1-3* represent a control experiment in which Rad51 was incubated with glutathione beads.

**
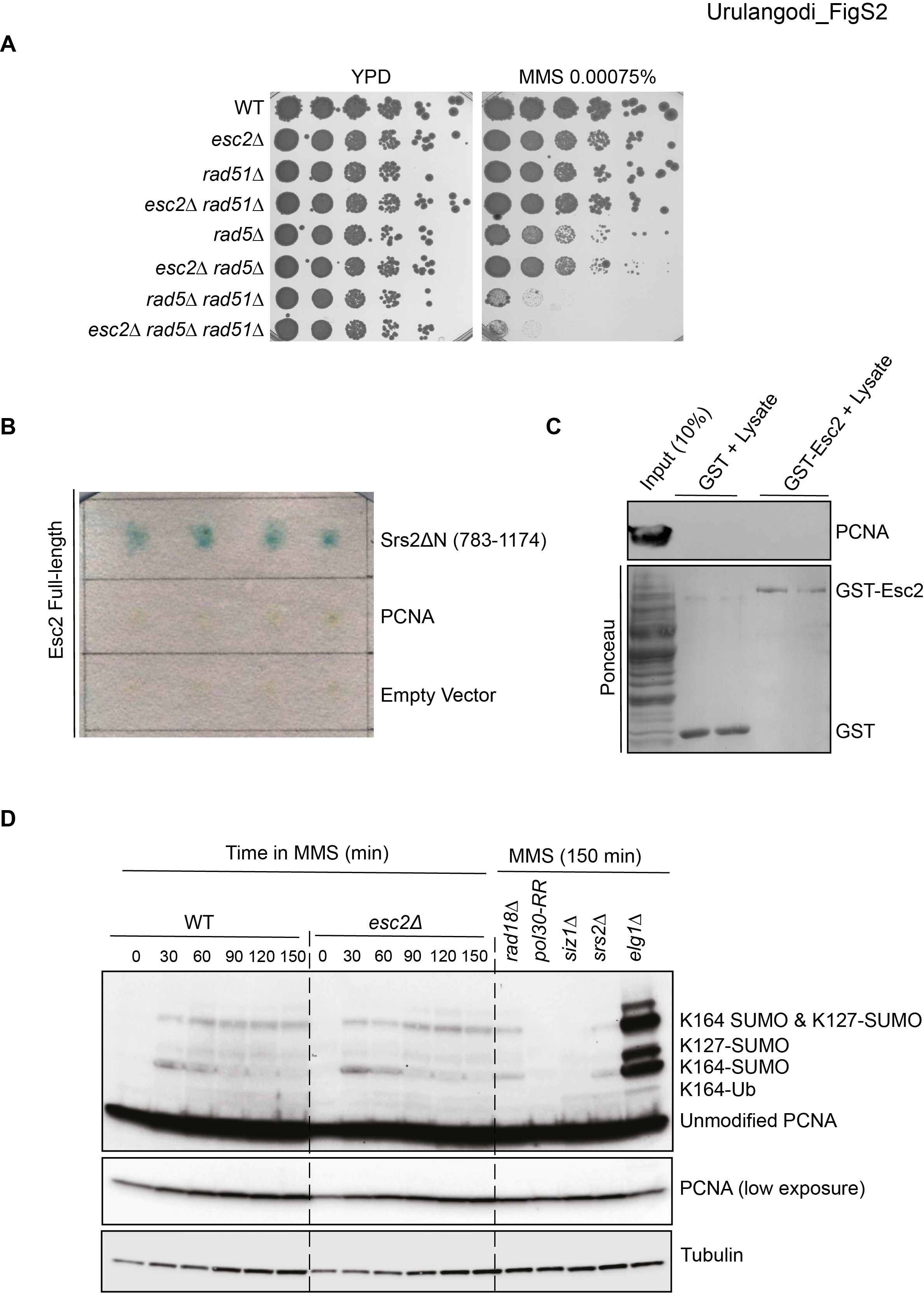
**

**Figure S2** Genetic and physical interaction of Esc2 with factors and modulators of the error-free DDT pathway. *(A)* WT, *esc2Δ*, *rad51Δ, esc2Δ rad51Δ*, *rad5Δ*, *esc2Δ* *rad5Δ*, *rad5Δ rad51Δ*) and *esc2Δ* *rad5Δ rad51Δ* strains were analyzed for MMS sensitivity by spot assay. *(B)* Esc2 does not physically interact with PCNA. Interaction examined by β-galactosidase assay in a yeast 2-hybrid assay system. Srs2ΔN (783-1174) was used as positive control (Pfander et al. 2005), and empty vector to control the specificity of detected interaction. *(C) In vivo* pull-down assay using GST-Esc2 and WT cell extracts. GST pull-down experiment was performed as described in Figure 2C and analyzed using an anti-PCNA antibody. Ponceau S-stained lower panel serves as loading control. *(D)* Western blot analysis of PCNA modifications after MMS treatment. Cells were released from G1 arrest into media containing 0.03% MMS. Samples were collected at the indicated time points and analyzed by western blot using an anti-PCNA antibody. Tubulin staining and low-exposed unmodified PCNA were used as loading control. Positions of each Ubiquitin and SUMO modifications were determined by comparing with various control strains (*rad18Δ*, *pol30-RR*, *siz1Δ*, *srs2Δ*, and *elg1Δ*)*.*

**
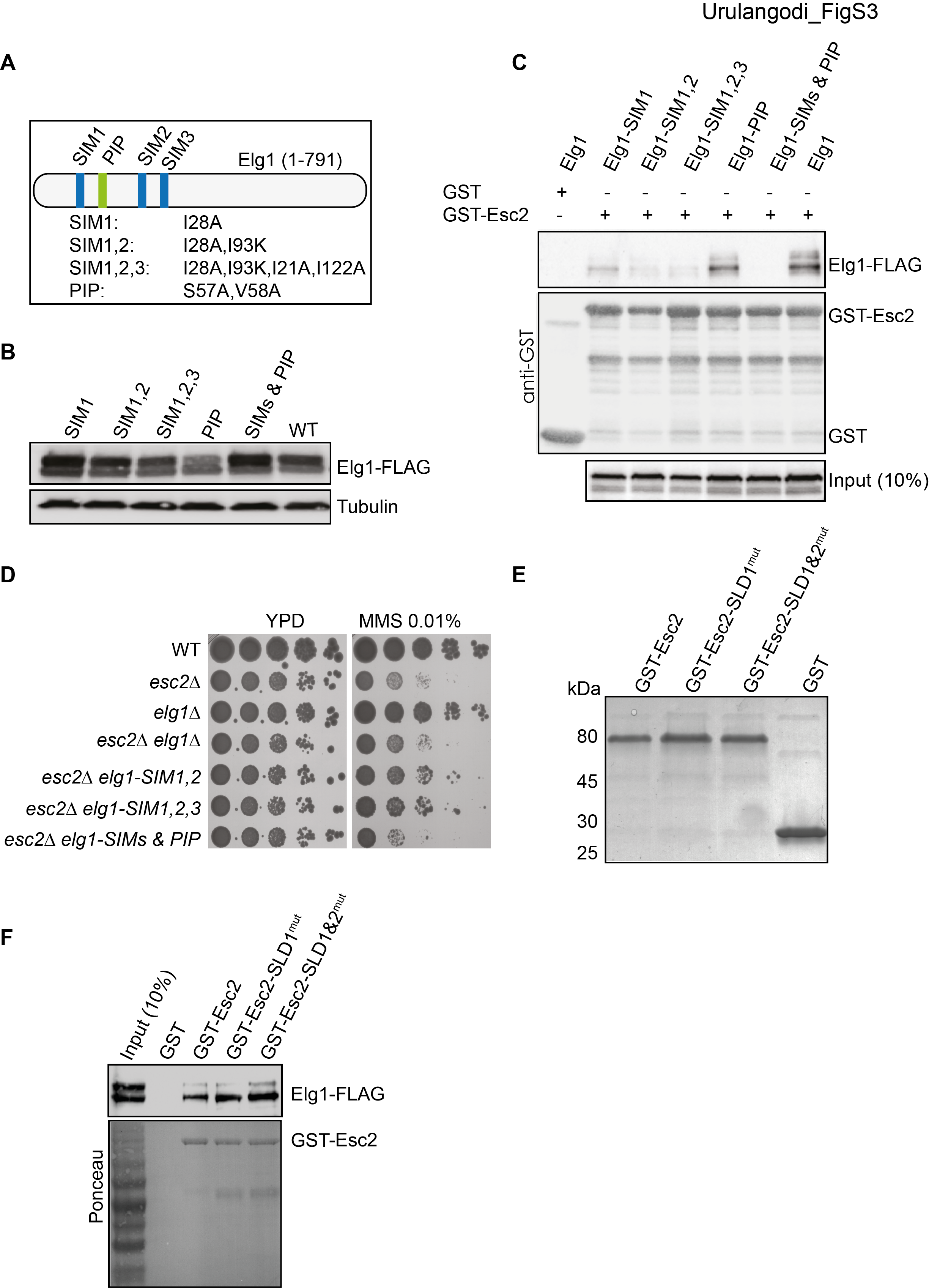
**

**Figure S3** Interaction of Elg1 with the SLD protein Esc2 is mediated by the SIM containing N-terminal region. *(A)* Schematic map of various Elg1 point mutants used for pull-down assay. *(B)* Elg1-SIMs and PIP mutant protein levels. Logarithmically grown WT and various *elg1* mutant strains were used to analyze the level of encoded FLAG-tagged Elg1 variants by western blot using an anti-FLAG antibody. Tubulin staining was used as loading control. *(C)* Pull-down using total cell lysates made from cells expressing FLAG-tagged *elg1* alleles with individual or combined mutations in SIMs and/or PIP. *(D)* Strains of the indicated genotype were analyzed for MMS sensitivity by spot assay. *(E)* Coomassie-stained samples of purified recombinant WT Esc2 and mutant proteins used for interaction studies. *(F)* Elg1 is proficient in interacting with Esc2*-*SLD1&2^mut^ variant. GST pull-down experiment was performed as described in Figure 3D.

**
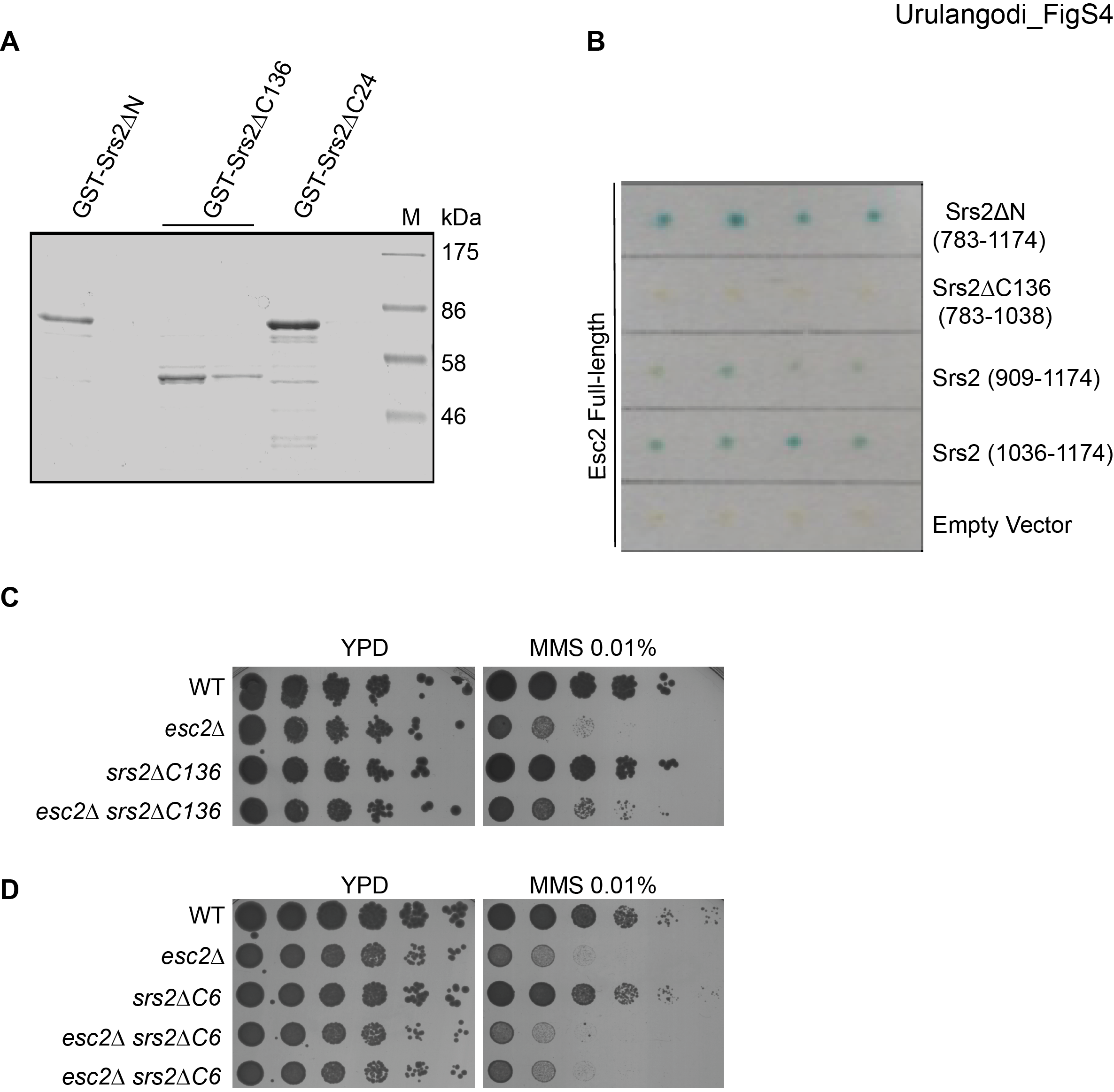
**

**Figure S4** SUMO Interacting Motifs **(**SIM) of Srs2 mediates interaction with Esc2**.** *(A)* Coomassie-stained samples of purified Srs2 mutant proteins used for GST-pull down assays. *(B)* The C-terminal domain of Srs2 interacts with Esc2 in a yeast 2-hybrid assay system. *(C-D)* Strains of the indicated genotype were analyzed for MMS sensitivity by spot assay.

**
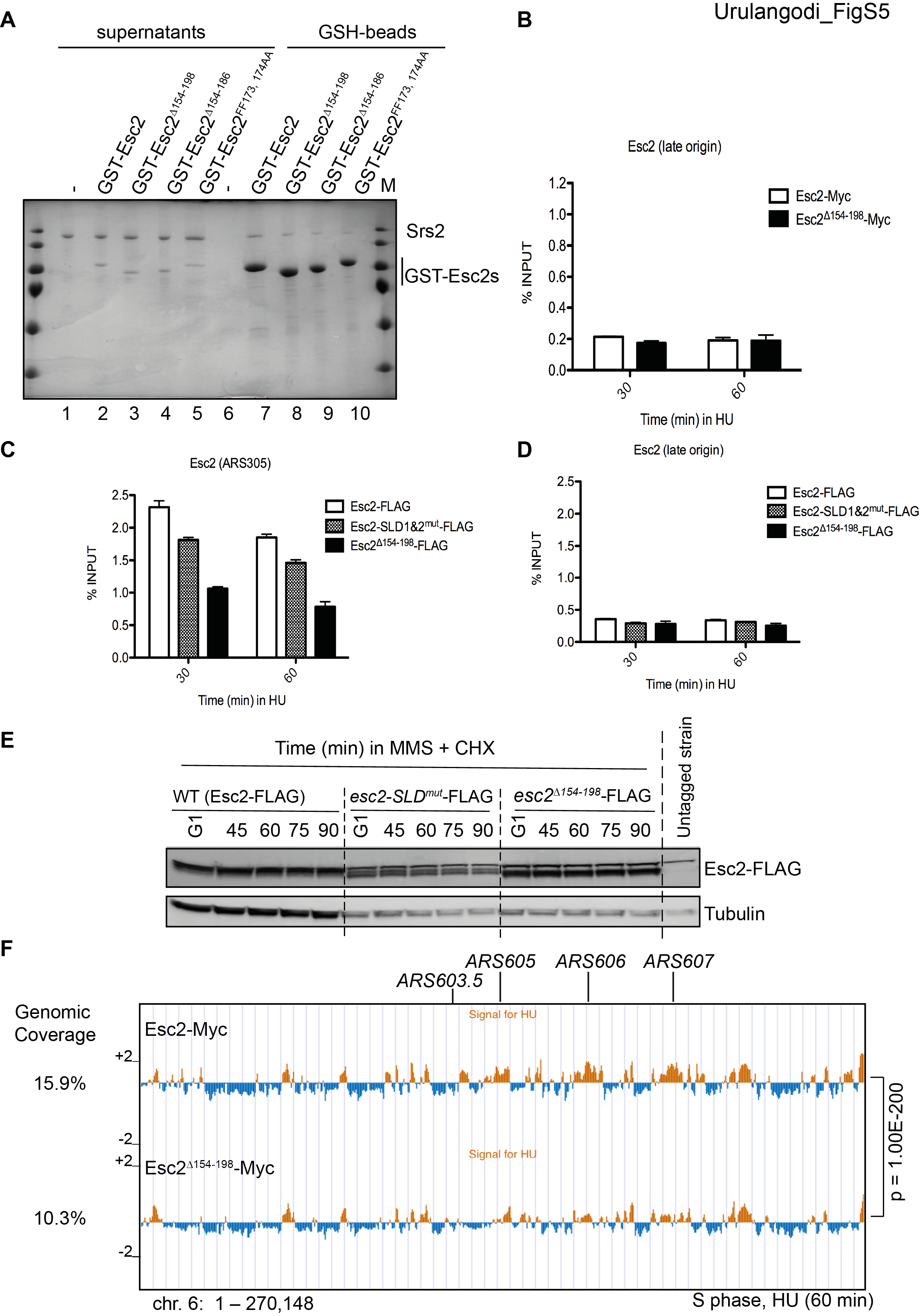
**

**Figure S5** Esc2^Δ154-198^ mutant is proficient in interacting with Srs2 and for chromatin association. *(A)* GST-Esc2^Δ154-198^ interacts with Srs2 in an *in vitro* pull down assay. *(B)* ChIP-qPCR for recruitment of Esc2-Myc and Esc2^Δ154-198^-Myc to the late/dormant origin of replication ARS440. The sample is same as in Figure 5B. *(C-D)* ChIP-qPCR for recruitment of Esc2-FLAG, Esc2^Δ154-198^-FLAG, and Esc2-SLD1&2^mut^-FLAG to the early origin ARS305 or the late/dormant origin of replication ARS440. *(E*) Stability of Esc2-FLAG, Esc2-SLD1&2^mut^-FLAG and Esc2^Δ154-198^-FLAG protein analyzed by cycloheximide (CHX) chase experiment. *(F)* Genome-wide binding pattern of Esc2 and Esc2^Δ154-198^ by ChIP-on-chip in 0.2 M HU. The experiment was performed and analyzed as described in Figure 4B. Chromosome 6 is shown as representative example.

**
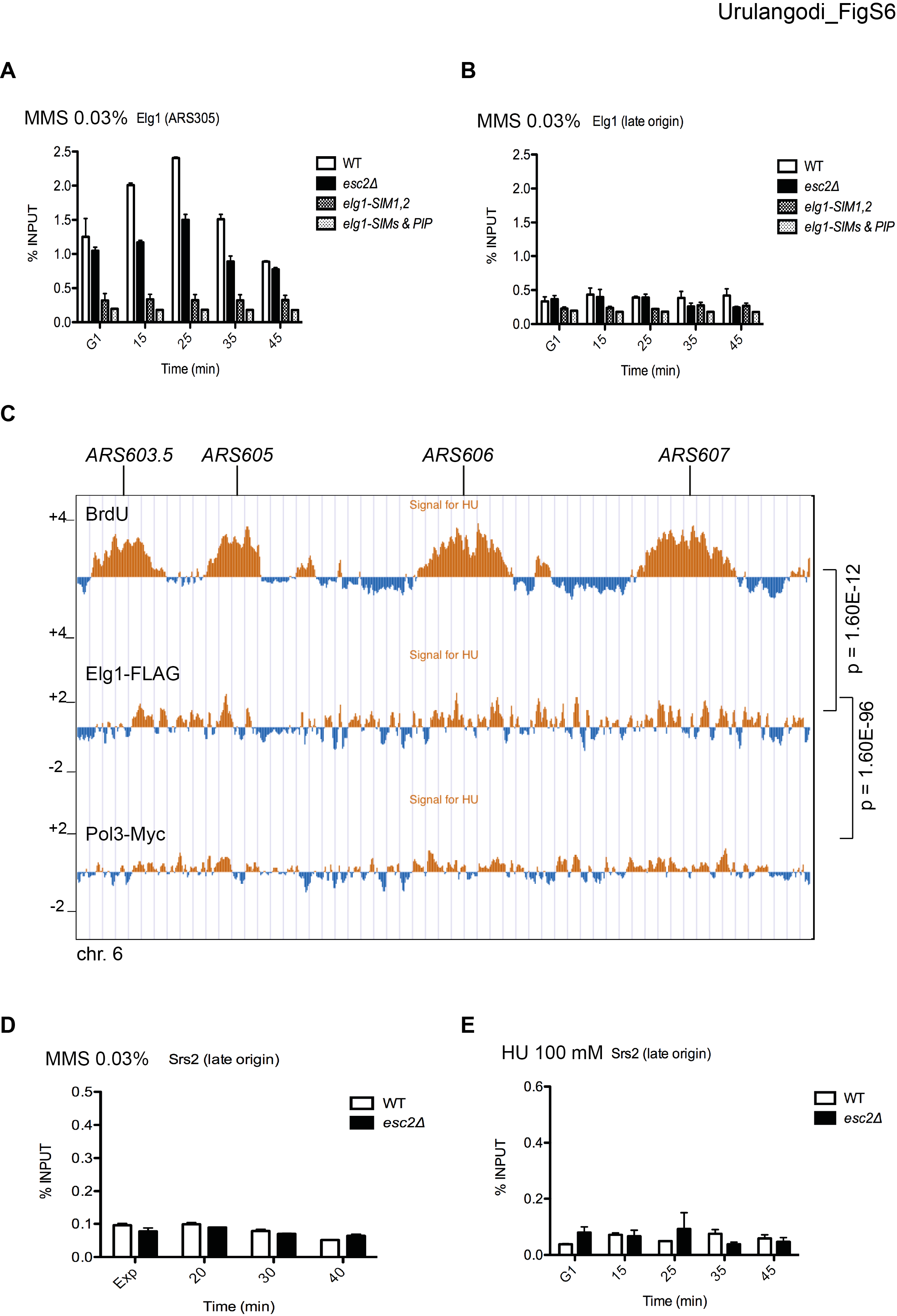
**

**Figure S6** Esc2 promotes Elg1 stable association to chromatin. *(A-B)* ChIP-qPCR assays to analyze the recruitment of FLAG epitope tagged Elg1, Elg1-SIM1,2, and Elg1-SIMs & PIP to the early origin of replication ARS305 (A) or to the dormant/late origin ARS440 in the presence of 0.03% MMS. *(C)* The genome-wide overlap between the binding clusters of Elg1 ChIP-on-chip with BrdU and Pol3. The Elg1 binding data is same as described in Figure 6C. The the p-values indicate the significance of overlap between the considered protein clusters. *(C-D)* Srs2 binding at late/dormant (ARS440) origins during replication in the presence 0.03% or 0.1 M HU. The samples were the same as in Figure 6D-E.

**
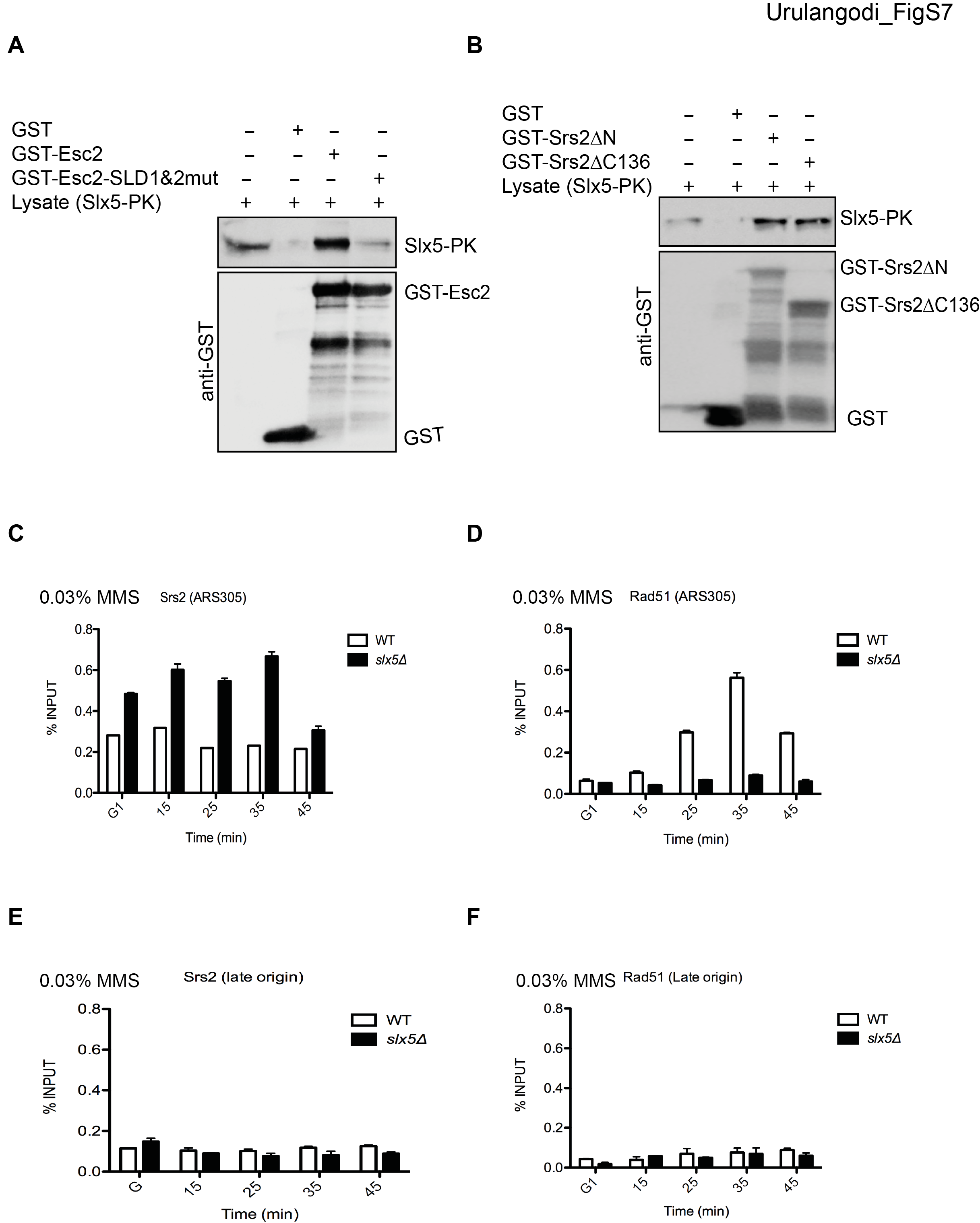
**

**Figure S7** Slx5 interacts with both Esc2 and Srs2. *(A)* *In vivo* pull-down assay using GST-Esc2. GST pull-down experiment was performed as described in Figures 2C-D using total lysates prepared from cells expressing Slx5-PK (V5). *(B)* GST pull-down assay using GST-Srs2ΔN and GST-Srs2ΔC136. The amounts of GST, GST- Esc2 and GST-Srs2ΔN and GST-Srs2ΔC136 proteins used are shown by immunoblot with anti-GST antibody. *(C-D)* ChIP-qPCR assay was performed to analyze the recruitment of Srs2 and Rad51 to early origins of replication (ARS305) in the presence of 0.03% MMS. *(E-F)* Srs2 and Rad51 recruitment to the dormant/late origin ARS440 by ChIP-qPCR.

**
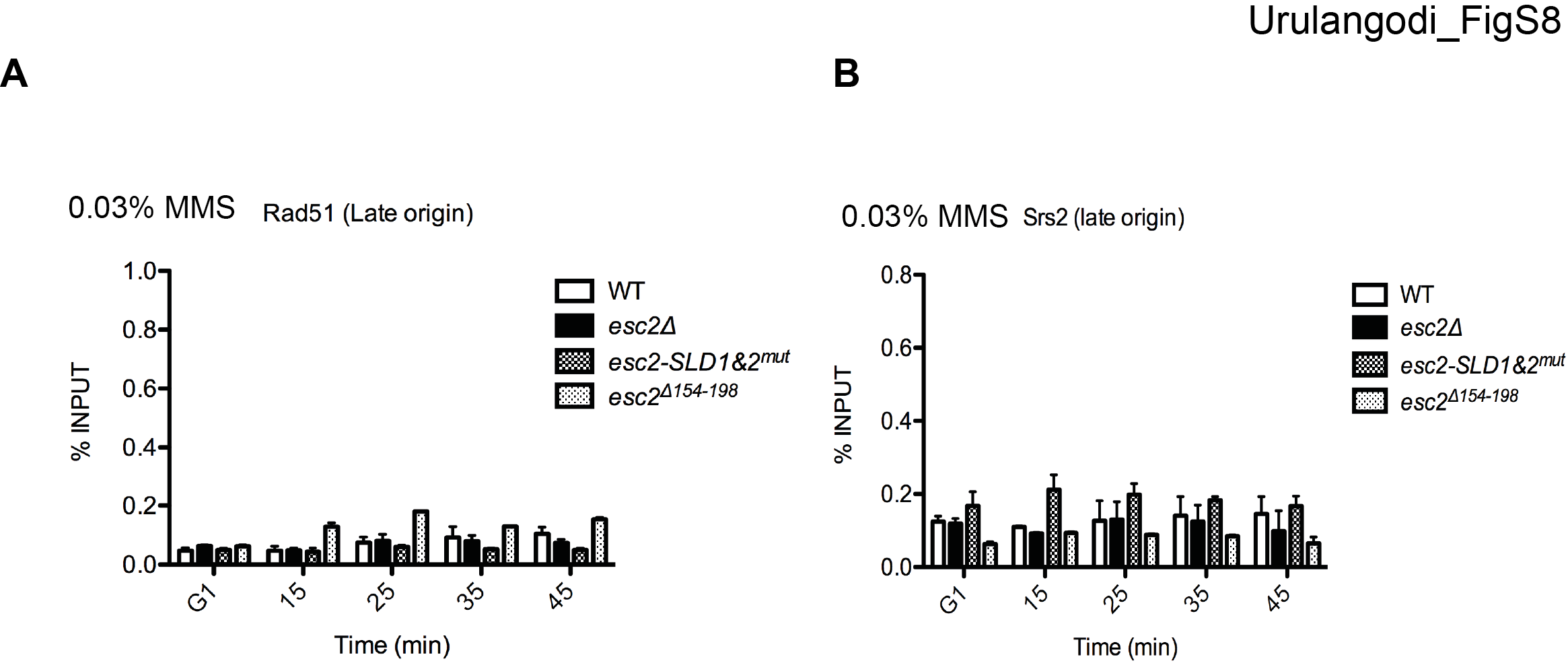
**

**Figure S8** The SLD and DNA binding domain *esc2* mutants are stably expressed and do not affect Rad51 and Srs2 recruitment at regions that do not contain stalled forks. *(A-B)* ChIP-qPCR assay was performed to analyze the recruitment of Rad51 and Srs2 to the dormant/late origin ARS440 in the presence of 0.03% MMS. The samples were the same as in Figure 7A-B.

**
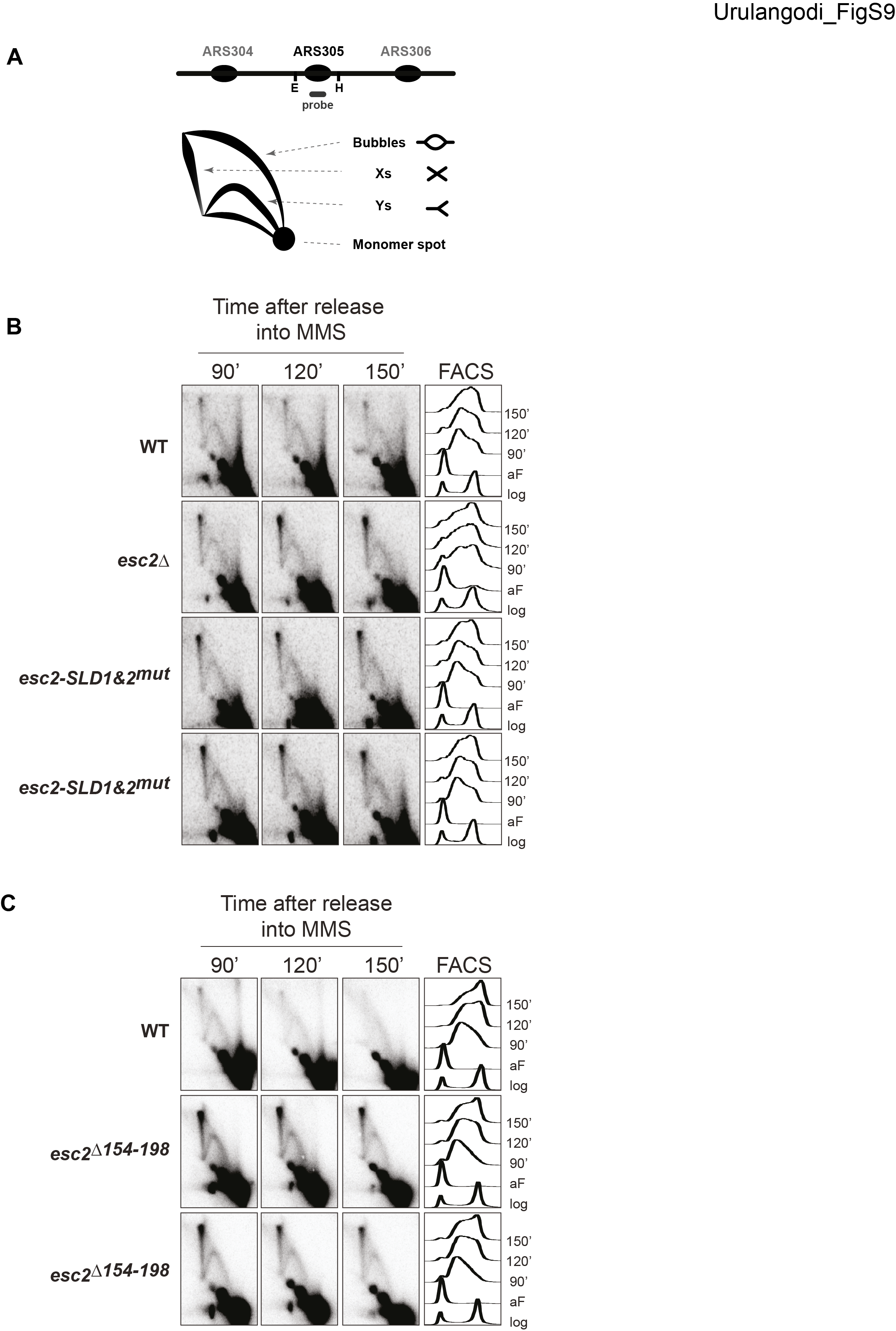
**

**Figure S9 The** SLD and DNA binding domain *esc2* mutants accumulate X-molecules during replication of damaged templates. *(A)* Schematic representation of 2D gel intermediates with the analyzed region and probe. *(B-C)* 2D gel profiles of replication intermediates isolated from cells of the indicated genotype. Cells were released from G1 arrest in media containing MMS 0.033% and samples for 2D gel and FACS analysis were collected at the indicated time points. Two independent clones were tested for *esc2-SLD1&2^mut^* and *esc2^Δ154-198^*.

**Table S1:** Yeast strains used in this study

| Strain | Genotype | Source |
| --- | --- | --- |
| FY1000  (W303 wt) | MATa *ade2-1 can1-100 his3-11-15 leu2-3, 112 trp1-1 ura3-1 RAD5+* | Lab collection |
| FY1001 | W303 MATa *esc2Δ::KANMX* | Lab collection |
| FY1002 | W303 MATa *rad51Δ::LEU2* | Lab collection |
| FY1017 | W303 MATa *slx8Δ::KANMX* | Lab collection |
| FY1026 | W303 MATα *esc2Δ::NATMX* | Sollier *et al*., 2009 |
| FY1028 | W303 MATa *ESC2-13MYC::KANMX* | Sollier *et al*., 2009 |
| FY1081 | W303 MATa *esc2Δ::NATMX* | Lab collection |
| FY0112 | DF5a MATa *pol30-K164R,K127R* | Lab collection |
| FY1110 | Mat a *ura3::URA3/GPD-TK(7X)* | Lab collection |
| FY0050 | W303 MATa *srs2::3HA-SRS2* | Lab collection |
| FY1624 | *Mat a Pol3-9Myc::TRP1* | Lab collection |
| FY1805 | MATα *cim3-1*, *ura3-52, leu2-delta1* | Lab collection |
| FY1846 | W303 *Slx5-V5::TRP1* | Lab collection |
| HY0121 | W303 MATa *rad18Δ::HPHMX* | Lab collection |
| HY0628 | W303 MATa *siz1Δ::HPHMX* | Lab collection |
| HY0698 | W303 MATa *rad5Δ::HPHMX* | Lab collection |
| HY1111 | W303 MATa *esc2Δ::HIS3* | Sollier *et al*., 2009 |
| HY1362 | W303 MATa *rad5Δ::HPHMX esc2Δ::HIS3* | Lab collection |
| HY1976 | W303 MATa *Elg1-10 FLAG::KANMX* | This study |
| HY1978 | W303 MATa *elg1Δ::HIS* | This study |
| HY2003 | W303 MATa *elg1Δ::KANMX* | This study |
| HY2009 | W303 MATa *esc2Δ::NATMX::Elg1-10FLAG::KANMX* | This study |
| HY2014 | W303 MATa *esc2Δ::NATMX elg1Δ::KANMX* | This study |
| HY2786 | W303 MATa *slx5Δ::HIS3* | This study |
| HY3344 | W303 MATa *esc2Δ::HIS3 rad51Δ::LEU2* | This study |
| HY3364 | W303 MATa *Elg1(SIM1)-10FLAG::KANMX* | This study |
| HY3366 | W303 MATa *Elg1(SIM1,2)-10FLAG::KANMX* | This study |
| HY3368 | W303 MATa *Elg1(SIM1,2,3)-10FLAG::KANMX* | This study |
| HY3369 | W303 MATa *Elg1(PIP)-10FLAG::KANMX* | This study |
| HY3371 | W303 MATa *Elg1(SIMs and PIP)-10 FLAG::KANMX* | This study |
| HY3591 | W303 MATa *esc2Δ::NATMX Elg1(SIM1,2)-10FLAG::KANMX* | This study |
| HY3596 | W303 MATa *esc2Δ::NATMX Elg1(SIM1,2,3)-10FLAG::KANMX* | This study |
| HY3605 | W303 MATα *Elg1(SIMs and PIP)-10 FLAG::KANMX* | This study |
| HY3606 | W303 MATa *esc2Δ::NATMX Elg1(SIMs and PIP)-10 FLAG::KANMX* | This study |
| HY3692 | W303 MATa *rad5Δ::HPHMX rad51Δ::LEU2* | This study |
| HY3694 | W303 MATa *esc2Δ::HIS3 rad5Δ::HPHMX rad51Δ::LEU2* | This study |
| HY4523 | W303 MATa *Esc2-10 FLAG::KANMX* | This study |
| HY5329 | W303 MATa *pdr5Δ::HIS3* | This study |
| HY5607 | W303 MATa *Esc2-SLD1&2^mut^-10FLAG::KANMX* | This study |
| HY5634 | W303 MATa *srs2delC6-HA::HIS3* | This study |
| HY5636 | W303 MATa *esc2Δ::NATMX srs2delC6-HA::HIS3* | This study |
| HY5640 | W303 MATa *Srs2ΔC136-HA::HIS3* | This study |
| HY5642 | W303 MATa *esc2Δ::NATMX Srs2ΔC136-HA::HIS3* | This study |
| HY5874 | W303 MATa *srs2::3HA-SRS2 esc2Δ::HIS3* | This study |
| HY5923 | W303 MATa *esc2^Δ154-198^-13Myc::KANMX4* | This study |
| HY5934 | W303 MATa *srs2::3HA-SRS2*  *esc2^Δ154-198^-10FLAG::KANMX* | This study |
| HY5938 | W303 MATa *srs2::3HA-SRS2*  *slx5Δ::HIS3* | This study |

**Table S2**: Oligonucleotides used in this study.

| Number | Name | Sequence (5’-3’) |
| --- | --- | --- |
| 1 | Elg1_128A_up | AAGATGCTCTACAGATTACCGCCGATGACGAAAATGATACTGA |
| 2 | Elg1_I28A_down | TCAGTATCATTTTCGTCATCGGCGGTAATCTGTAGAGCATCTT |
| 3 | Elg1_I93K_up | ATGACGACGATGATGATCTTAAAGTAATCAGTGATAAGAGTCC |
| 4 | Elg1_I93K_down | GGACTCTTATCACTGATTACTTTAAGATCATCATCGTCGTCAT |
| 5 | Elg1_II121,122AA_up | AGCATGAAGATGATATTTCTGCCGCTTCCACATCGAGAATCAAATC |
| 6 | Elg1_II121,122AA_down | GATTTGATTCTCGATGTGGAAGCGGCAGAAATATCATCTTCATGCT |
| 7 | Elg1_SV57,58AA_down | ACAGCTTCAATTGGCTTCACAGCAGCGTGGTTTAAAAATATTACTG |
| 8 | Elg1_FP2 | ATGAAAAGGCACGTGTCTTTATCTGATA |
| 9 | Elg1_RP2 | GCCAAGTAATACCGGTGCTTGATAG |
| 10 | Elg1_FP3 | GATCGGAGTGTTGATATCCCGCTTCCTTTCCGCACAATACCGC |
| 11 | Elg1_RP3 | CATACGTGTTCCTGTAACGATGCACGCAATTTCATATCAGTG |
| 12 | Elg1_FP4 | GTACCATAGCATTAAACTTTTTGAATCAGAGAAGGTTTTCCAATGAAAAGGCACGTGTCTTTATCTG |
| 13 | Elg1_FLN2 | GAATAGCTGCGGCCATGTGAGAGTTGTATTTCTTTTAATATTTGAACTATAGGGAGACCGGCAGATC |
| 14 | ARS305F | CTCCGTTTTTAGCCCCCCGTG |
| 15 | ARS305R | GATTGAGGCCACAGCAAGACCG |
| 16 | trs31F | CGAAAGTGACGAAGTTCATGC |
| 17 | trs31R | GCCATTGCTGATAAAGACGC |
| 18 | *5’-Fluorescein labeled oligo | *AGCTACCATGCCTGCACGAATTAAGCAATTCGTAATCATGGTCATAGCT |
| 19 |  | AGCTATGACCATGATTACGAATTGCTTAATTCGTGCAGGCATGGTAGCT |
| 20 |  | CTACAGTTCGTCAGGATTCC |
| 21 |  | AATTCGTGCAGGCATGGTAGCT |
| 22 |  | AGCTATGACCATGATTACGAATTGCTTGGAATCCTGACGAACTGTAG |
| 23 | Esc2_D286A_Top | GAAGTCTCTGCTATCGATGTTAC |
| 24 | Esc2_D286A_Bottom | GTAACATCGTAAGCAGAGACTTC |
| 25 | Esc2_D447AD449A_Top | GGATATGGAAGCTGAAGCCATGGTTGAT GTC |
| 26 | Esc2_D447AD449A_Bottom | GACATCAACCATGGCTTCAGCTTCCATA TCC |
| 27 | Esc2F1 | ATGACCGGTGATTCCAGAAGC |
| 28 | Esc2R1 | GCCTTTTTTAGCAAATTGCGC |
| 29 | Esc2 faFP1 | CAATAAAAGGCAAGAGAAAAAATCATTT GTTTTAGGCTGTTAACAGATGACCGGTG ATTCCAGAAGC |
| 30 | Esc2 faRP1 | CGCCCATAGTAAATAATAATTGTGGCAC GTAAAGAGCCGGAGTTCGGCCTTTTTTA GCAAATTGCGC |
| 31 | ESC2^Δ154-198^F | GCATAAGATCAATATCTCCAGCGCGAGTTTATAACATCAAATTTCTCTCTAAGC |
| 32 | ESC2^Δ154-198^R | GAAATTTGATGTTATAAACTCGCGCTGGAGATATTGATCTTATGCTTGACCTGG |
| 33 | ESC2^Δ154-186^F | GCATAAGATCAATATCTCCAGCGATTTCTAAAGAATCGACACCCGACCAGCG |
| 34 | ESC2^Δ154-186^R | CGGGTGTCGATTCTTTAGAAATCGCTGGAGATATTGATCTTATGCTTGACCTGG |
| 35 | Esc2^FF173, 174AA^F | CTGTATACCTATGATGAAAACGATGACGCTGCCAAGGAACTTGCTAAAGAAGCCAAAAAA |
| 36 | Esc2^FF173, 174AA^R | TTTTTTGGCTTCTTTAGCAAGTTCCTTGGCAGCGTCATCGTTTTCATCATAGGTATACAG |
| 37 | Esc2 RF1 | CTTCATCAATCCGTCCAAAGC |
| 38 | Esc2 aFP1 | CTCTCTAAGCTAGAAGGAAC |
| 39 | Esc2^1-199^F | GGGGATCCATGACCGGTGATTCCAGAAGC |
| 40 | Esc2^1-199^R | TTACCCGGGAACTCGCTTCCGCTGGT |
| 41 | Esc2^1-294^F | CTATCGTATCCAAAGAATACTGAAAGAACTTTGAAGCGACACTGG |
| 42 | Esc2^1-294^R | CCAGTGTCGCTTCAAAGTTCTTTCAGTATTCTTTGGATACGATAG |
| 43 | Esc2^1-374^F | GCAAGAGGGTGGCTCTTTGTCATAGAATAGTAGTAGCATGGAAGAAG |
| 44 | Esc2^1-374^R | CTTCTTCCATGCTACTACTATTCTATGACAAAGAGCCACCCTCTTGC |
| 45 | Esc2^200-456^F | GCGAATTCTATAACATCAAATTTCTCTC |
| 46 | Esc2^200-456^R | CGTTGTCGACTCAATCAATAATGACATC |

**Materials and methods**

*Yeast strains and plasmids*

The strains used in this study are listed in the Supplementary Table S1. In case of tagged strains, the epitope tags (FLAG, MYC) were fused to the C-terminus of the protein of interest. The *elg1* mutant alleles, *elg1-I28A* (SIM1), *elg1-I28A, I93K (SIM1,2)*, e*lg1-I28A, I93K, II121,122AA (SIM1,2,3)*, *elg1-SV57,58AA (PIP)*, and *elg1- I28A, I93K, II121,122AA, SV57,58AA* (SIMs & PIP), were generated using Quick Change Site-Directed kit (Stratagene) to modify *ELG1****,*** subcloned at BamH1-Sal1 restriction site in the pGEX-6P-2 (HB104) vector. The sequences of the oligonucleotides used are listed in Supplementary Table S2 (oligos 1-7). Integration of *elg1* alleles (SIM and PIP mutants) at the *S. cerevisiae ELG1* chromosomal locus was based on a three-step PCR-based strategy, largely as described in ([Xiong et al. 2006](#_ENREF_7)). Specifically, the 1-690 bp fragment of Elg1 N-terminus carrying the respective mutations were amplified from corresponding mutagenized plasmids using the primers FP2 and RP2 (Supplementary Table S2). A second set of PCR was performed to amplify the remaining Elg1 fragment with C-terminal FLAG tag and the *KanMX* selection marker (≈ 3.8 kb) using a 55 nt overlapping primers (FP3 and RP3, Supplementary Table S2) from the yeast strain carrying the Elg1-10FLAG::*KanMX4* allele integrated at the *ELG1* locus (HY2009, Supplementary Table S1). The amplified PCR products were gel purified and used for a third round of PCR to amplify the mutant full-length *elg1-10FLAG* cassettes using the primers FP4 and FL N2 (Supplementary Table S2). These individual PCR products (≈ 4.5 kb) were then transformed in *elg1Δ*::*HisMX6* (HY1978, Supplementary Table 1). The mutants were selected on Geneticin G418 containing plates and positive clones were tested for their lack of growth on -His selection plates. The mutations were then confirmed by DNA sequencing. Similarly, *esc2* mutant alleles, *esc2-D226A, D227Y* (SLD1^mut^*)*, and *esc2-D447A, D449A* (SLD2^mut^), were generated by site-directed mutagenesis using the oligonucleotides listed in Supplementary Table S2 (oligos 23-26). A similar strategy described above was employed to integrate Esc2-SLD1&2^mut^ (D286A, I287Y, D447A, D449A) with C-terminal 10FLAG tag using the primers 27-30 (Supplementary Table S2). Internal deletion mutants of Esc2 (Esc2^Δ154-198^ and Esc2^Δ154-186^) were generated by a PCR-based method described in ([Hansson et al. 2008](#_ENREF_2)) using the oligonucleotides listed in Supplementary Table S2 (oligos 31-34) and pGEX-6p1-Esc2 as a template. The Esc2-F173A, F174A mutant was generated using Quick Change Site-Directed kit (Stratagene). Integration of these *esc2* alleles at the *S. cerevisiae* Esc2 chromosomal locus was performed as described above. The N-terminal fragment of *Esc2* carrying the respective deletions or mutations were amplified from corresponding mutagenized plasmids using the primers Esc2 F1 and Esc2 RF1 (Supplementary Table S2). A second set of PCR was performed to amplify the remaining Esc2 fragment with C-terminal FLAG or Myc tag and the *KanMX* selection marker (≈ 2.2 kb) using 55 nt overlapping primers (Esc2 R1 and Esc2 aFP1, Supplementary Table S2) from the yeast strain carrying either Esc2-10FLAG::*KanMX4* (HY4523) or Esc2-13Myc::*KanMX4* (FY1028) allele integrated at the *esc2Δ* locus. The amplified PCR products were gel purified and used for a third round of PCR to amplify the mutant full-length *esc2-10FLAG or esc2-13Myc* cassettes using the primers Esc2 faFP1 and Esc2 faRP1 (Supplementary Table S2). These individual PCR products were then transformed in *esc2Δ*::*NATMX* (FY1081). The mutants were selected on Geneticin G418 containing plates and positive clones were tested for their lack of growth on selection plates containing NAT. The mutations were then confirmed by DNA sequencing.

Expression vectors for GST-Srs2 and their truncated variants were based on the pGEX-6P-2 plasmid (GE Healthcare). To construct GST-Esc2, the full-length Esc2 gene amplified from *S. cerevisiae* genomic DNA was cloned into the BamH1-Sal1 sites of pGEX-6P-2 vector. The GST-Esc2 truncation variants (Esc2^1-199^, Esc2^1-294^ and Esc2^1-374^) were generated by site-directed mutagenesis of the plasmid pGEX-6P-2-Esc2, introducing a STOP codon at positions corresponding to aminoacids Y200, E295 and G375, respectively using the primers listed in Supplementary Table S2 (oligos 39-44). Esc2^200-456^ was generated by PCR amplification of DNA fragment containing the corresponding DNA sequence cloned into EcoRI and SalI sites of pGEX6-P1, yielding plasmids pGEX6-P1-Esc2^200-456^ (oligos 45-46, Supplementary Table S2).

The GST-Srs2 783-1174 (pGEX4T1-Srs2ΔN) construct was received from S. Jentsch and described in ([Pfander et al. 2005](#_ENREF_5)). The His-Srs2 construct was described in ([Colavito et al. 2009](#_ENREF_1)). The Srs2ΔN fragment amplified from pGEX4T1-Srs2ΔN was sub-cloned into the pGEX-6P-2 vector at the BamH1 and SalI restriction sites to obtain a PreScission Protease cleavage site between GST and Srs2ΔN (HB097). Srs2ΔC136 (783-1038, HB098) and Srs2ΔC24 (783-1038, HB099) were generated by sub-cloning the respective PCR fragments into the BamH1-Sal1 sites of pGEX-6P-2 vector.

The two-hybrid constructs used in this study were described in ([Sollier et al. 2009](#_ENREF_6)), except for pGADC2-SRS2ΔN (783-1174), pGADC2-SRS2-783-1038, pGADC1-SRS2-909-1174, pGADC1-SRS2-1036-1174 that were received from S. Jentsch and described in (Pfander et al. 2005).

*Purification of recombinant proteins*

The *Esc2* and its truncated forms were expressed as a GST fusion proteins in *E. coli* BL21 RIPL cells (induction: 30ºC, 0.5 mM IPTG, 3 h). The cell pellets were sonicated in 50 ml of lysis buffer C (50 mM Tris-HCl, 10 % sucrose (w/v), protease inhibitors (aprotinin, chymostatin, leupeptin, pepstatin A, benzamidine, each at 5 μg/ml), 10 mM EDTA, 1 mM dithiothreitol (DTT), 0.01% (v/v) Nonidet-P40, and 100 mM KCl, pH 7.5). The crude lysate was clarified by centrifugation (100,000xg for 60 min). The supernatant was loaded onto a 10-ml Q sepharose column (GE Healthcare Life Sciences) equilibrated with buffer T (25 mM Tris-Cl, 10 % (v/v) glycerol, 5 mM EDTA, pH 7.5) containing 100 mM KCl and eluted with a 150-ml linear gradient of 100-500 mM KCl in buffer T. The peak fractions were pooled and batched for 1 h with 2 ml of glutathione-sepharose 4B beads (GE Healthcare Life Sciences) equilibrated with buffer T containing 150 mM KCl. GST-Esc2 was eluted with 6 x 2 ml of 20 mM glutathione in buffer T supplemented with 150 mM KCl. After the elution, GST-Esc2 fractions were pooled and split into two aliquots. The first aliquot was diluted with 5 ml of buffer T and loaded onto a 1-ml Mono Q column (GE Healthcare Life Sciences) equilibrated in buffer T containing 100 mM KCl. The GST-Esc2 fragments were eluted with a 20-ml gradient of 100-600 mM KCl in buffer T. Homogenous GST-Esc2 fractions were concentrated in a Vivaspin concentrator (Sartorius Stedim Biotech) and stored in 10-μl aliquots at −80°C. The second aliquot was incubated with 5-25 μg of PreScission protease for 3 hours at 4ºC, to cleave the GST-tag. Next, the sample was diluted with 5 ml of buffer T and loaded onto a 1-ml Mono Q column equilibrated in buffer T containing 100 mM KCl. Esc2 was eluted with a 20-ml gradient of 100-500 mM KCl in buffer T. Fractions containing homogenous Esc2 were concentrated in a Vivaspin concentrator (Sartorius Stedim Biotech) and stored in 10-μl aliquots at −80°C. GST-Esc2^193-294^ and GST-Esc2^193-374^ were expressed and purified as described above, with the exception that the initial Q-sepharose chromatographic step was omitted.

For GST-pull down assays, the proteins were purified as described below. *E. coli* BL21(DE3) cells carrying the pGEX-6P-2-Esc2 (full-length, truncated versions and mutants), or pGEX-6P-2-Srs2 truncation plasmid constructs were grown at 37°C in LB media containing Ampicillin to an *OD*600 of 0.6, and protein expression was induced by the addition of 0.5 mM IPTG. After 3 h of induction at 37°C, the culture was cooled on ice, and the cells were harvested by centrifugation and sonicated in PBST (Phosphate-buffered saline containing 1% Triton X-100) containing 10% glycerol and protease inhibitor cocktail (1:100, Calbiochem). The supernatant was collected and incubated with equilibrated glutathione-Sepharose 4B beads (Amersham Pharmacia Biotech) at 4°C for 3 hours. The beads were washed twice with PBST buffer and three times with Tris-HCl (pH 7.5) buffer containing 500 mM NaCl, and eluted with 20 mM reduced glutathione. The eluted proteins were dialyzed overnight at 4°C against storage buffer [20 mM Tris-HCl-pH 7.5, 140 mM NaCl, 1 mM DTT, 1 mM EDTA, and 10% glycerol] and divided into aliquots for storage at -80°C. Rad51 and full length Srs2 were purified as described before ([Colavito et al. 2009](#_ENREF_1)).

*GST in vivo pull-down assays using total yeast cell lysates*

Approximately 5 μg of GST or GST-fused protein were immobilized on 30 μl of glutathione-Sepharose 4B beads (GE healthcare). Total cell lysates were prepared from different yeast strains using a solubilizing buffer described below. Cells were arrested in G1 (α factor) and released into media with or without 0.03% MMS for 30 min. GST fusion proteins (5 μg) on glutathione beads were incubated with approximately 2.5 mg of yeast cell lysate at 4°C in Tris-HCl buffer (Tris pH 7.5, NaCl-150 mM, DTT-1mM, EDTA-1mM, Glycerol- 10%, Triton X-100 - 0.1%, and Protease inhibitor cocktail (1:100, Calbiochem) for 2-3 h either in presence or absence of Ethidium Bromide at a final concentration of 0.5 mg/ml. The beads were washed twice with Tris-HCl buffer and then twice with Tris-Buffer containing NaCl at 350 mM (for Srs2, PCNA and Slx5 pull-down) or 500 mM (for Elg1 pull-down) concentrations. The protein complexes isolated on the beads were eluted with 30 μl of 2xSDS Laemmli buffer and subjected to 10% SDS-PAGE for analysis by immunoblotting using antibodies against proteins or epitopes of interest. The proteins were then visualized by enhanced chemiluminescence (ECL), according to the manufacturer's instructions (Amersham ECL Plus).

*Yeast cell extract preparation*

Yeast native extracts for pull-down assays were prepared using liquid nitrogen described in ([Sollier et al. 2009](#_ENREF_6)). Briefly, a yeast cell pellet derived from 200 ml culture of about 1X10^7^ cells/ml was washed twice in 1X TBST buffer, and re-suspended in 5 ml of lysis buffer containing 50 mM HEPES pH 7.4, 140 mM NaCl, 1 mM EDTA, 1% Triton X-100, 0.1 % Na-deoxycholate supplemented with protease inhibitors cocktail (1:100, Calbiochem). The cells were lysed mechanically in liquid nitrogen using mortar and pestle. Supernatant fractions were collected following centrifugation at 12000 rpm for 30 min at 4°C, and the supernatant was re-spinned for 10 min at 4°C. Roughly 500 μL of lysate (corresponding to approximately 2.5 mg of total protein) was used for each pull down assay.

*In vitro pull-down assays*

To study the interaction between wild-type Srs2 and Esc2, purified His-tagged Srs2 (3 μg) was incubated with Esc2 (3 μg) and 10 μl of Ni-NTA beads (Qiagen) in 30 μl of buffer T (20 mM Tris-Cl, 10% glycerol, 1 mM EDTA, 1 mM DTT, 0.01% Nonidet P-40, pH 7.5) containing 100 mM KCl, for 30 min at 4°C, with gentle mixing. After the incubation, the supernatants were collected and mixed with 30 μl of 2xSDS Laemmli buffer. The beads were washed twice with 100 μl of the buffer T containing 100 mM KCl. Bound proteins were eluted with 30 μl of 2xSDS Laemmli buffer. The supernatant (S), wash (W) and SDS eluate (E) (10 μl each) were analyzed by SDS-PAGE on 10% gel followed by Coomassie Blue staining. Alternatively, GST-tagged Esc2 (3 μg) was incubated with Srs2 (3 μg) and 10 μl of Glutathione sepharose (GE Healthcare) in 30 μl of buffer T containing 100 mM KCl. Incubation and analysis was performed as described earlier. To study the interaction between Esc2 and Rad51, purified GST-Esc2 (5 μg) was incubated with Rad51 (5 μg) and 10 μl of Glutathione sepharose in 30 μl of buffer T containing 100 mM KCl. Incubation and analysis was performed as described earlier. To study the interaction between Srs2 C-terminal fragments and Esc2, purified GST-Srs2 C-terminal fragments (5 μg) were incubated with Esc2 (5 μg) and 10 μl of Glutathione sepharose in 30 μl of buffer T containing 100 mM KCl. Incubation and analysis was performed as described earlier. To study the interaction between Srs2 and Esc2 DNA binding domain mutants, purified GST-Esc2 and its fragments (3 μg) were incubated with the Srs2 (3 μg) in 30 μl of buffer T (20 mM Tris-HCl, pH 7.5, 100 mM KCl, 1 mM DTT, 0.5 mM EDTA, and 0.01% Nonidet P-40) for 30 min at 4°C in the presence of GSH-beads. After washing the beads twice with 100 μl of buffer T, the bound proteins were eluted with 30 μl of 5% SDS. The supernatant (S), wash (W), and SDS eluate (E), 10 μl each, were subjected to SDS-PAGE analysis.

*Electrophoretic Mobility Shift Assays (EMSA)*

Synthetic oligonucleotides were purchased from Sigma. The sequences of oligonucleotides used are listed in Supplementary Table S2 (oligos 18-22) and described in ([Matulova et al. 2009](#_ENREF_4)). The 5’-Fluorescein labeled oligonucleotide (oligo 18) was annealed with others to form dsDNA (oligos 18, 19), 3’-FLAP (oligos 18, 21, 22), and Fork (oligos 18, 20, 21, 22) structures. The substrates were prepared as described before ([Marini and Krejci 2012](#_ENREF_3)). Fluorescently-labeled DNA substrates (7 nM) were incubated with increasing concentrations of purified Esc2 at 37°C for 10 min in a reaction buffer containing 30 mM Tris (pH 7.5), 1 mM DTT, 5 mM MgCl2 and 20 mM KCl. The reactions were stopped by shifting the tubes onto ice followed by the addition of loading buffer. The reaction mixtures were resolved by electrophoresis on 7% native PAGE in 0.5 X TBE buffer. Gels were scanned using Typhoon Trio (GE Healthcare) or Fuji FLA 9000 imager with the Multi Gauge software (Fuji) and the quantified data were plotted using GraphPad Prism (version 5).

**References**

Colavito S, Macris-Kiss M, Seong C, Gleeson O, Greene EC, Klein HL, Krejci L, Sung P. 2009. Functional significance of the Rad51-Srs2 complex in Rad51 presynaptic filament disruption. *Nucleic acids research* 37: 6754-6764.

Hansson MD, Rzeznicka K, Rosenback M, Hansson M, Sirijovski N. 2008. PCR-mediated deletion of plasmid DNA. *Anal Biochem* **375**: 373-375.

Marini V, Krejci L. 2012. Unwinding of synthetic replication and recombination substrates by Srs2. *DNA repair* 11: 789-798.

Matulova P, Marini V, Burgess RC, Sisakova A, Kwon Y, Rothstein R, Sung P, Krejci L. 2009. Cooperativity of Mus81.Mms4 with Rad54 in the resolution of recombination and replication intermediates. *J Biol Chem* 284: 7733-7745.

Pfander B, Moldovan GL, Sacher M, Hoege C, Jentsch S. 2005. SUMO-modified PCNA recruits Srs2 to prevent recombination during S phase. *Nature* 436: 428-433.

Sollier J, Driscoll R, Castellucci F, Foiani M, Jackson SP, Branzei D. 2009. The Saccharomyces cerevisiae Esc2 and Smc5-6 proteins promote sister chromatid junction-mediated intra-S repair. *Molecular biology of the cell* 20: 1671-1682.

Xiong AS, Yao QH, Peng RH, Duan H, Li X, Fan HQ, Cheng ZM, Li Y. 2006. PCR-based accurate synthesis of long DNA sequences. *Nature protocols* 1: 791-797.
